# Inhibition of *Listeria monocytogenes* in White-Brined Cheese by *Lactobacillus reuteri*: Effect of *L. reuteri* Delivery Method, Brine Concentration, and Storage Temperature

**DOI:** 10.3390/foods14223964

**Published:** 2025-11-19

**Authors:** Amin N. Olaimat, Fajer Al-aittan, Murad Al-Holy, Anas Al-Nabulsi, Renad Haddad, Hamzah Al-Qadiri, Tareq Osaili, Mahmoud Abughoush

**Affiliations:** 1 Department of Clinical Nutrition and Dietetics, Faculty of Applied Medical Sciences, The Hashemite University, Zarqa 13133, Jordan; fajeralaitan@gmail.com (F.A.-a.); renad.haddad98@gmail.com (R.H.); 2 Department of Nutrition and Integrative Health, Faculty of Allied Medical Sciences, Middle East University, Amman 11831, Jordan; 3 Department of Nutrition and Food Technology, Faculty of Agriculture, Jordan University of Science and Technology, Irbid 22110, Jordan; 4 Department of Nutrition and Food Technology, School of Agriculture, University of Jordan, Amman 11942, Jordan; h.qadiri@ju.edu.jo; 5 Department of Clinical Nutrition and Dietetics, College of Health Sciences, University of Sharjah, Sharjah P.O. Box 27272, United Arab Emirates; 6 Science of Nutrition and Dietetics Program, College of Pharmacy, Al Ain University, Abu Dhabi P.O. Box 64141, United Arab Emirates; mahmoud.abughoush@aau.ac.ae

**Keywords:** *Lactobacillus reuteri*, *Listeria monocytogenes*, white-brined cheese, bio-preservation, reuterin, dairy products, brine solution

## Abstract

This study investigated the inhibitory effect of *Lactobacillus reuteri* with glycerol inoculated at different production stages of white-brined cheese (WBC) immersed in different concentrations of brine solutions under different storage temperatures as a bio-preservative against *Listeria monocytogenes*. Additionally, the physicochemical properties of WBC and brine solution were evaluated. A cocktail of five *L. reuteri* strains (~6 log CFU/g) with the addition of 100 mM glycerol was inoculated at either (1) water used to make the brine, (2) directly to the brine solution, or (3) pasteurized milk used to prepare cheese. The effect of *L. reuteri* against a cocktail of five *L. monocytogenes* strains (~5 log CFU/g) in WBC and stored in 10% or 15% brine at 4, 10, or 24 °C for 91 days was investigated. The salt content, pH, and water activity (a_w_) of WBC were also evaluated. *L. reuteri* inoculated in brine solution reduced the numbers of *L. monocytogenes* by 0.7–1.4 and 0.4–1.6 log CFU/g, in WBC and brines (10–15%), respectively, stored at different storage temperatures for 91 days compared to *L. monocytogenes* numbers in the absence of *L. reuteri* (control). When *L. reuteri* and glycerol were added to pasteurized milk during the production of WBC, the *L. monocytogenes* counts decreased by 1.2–2.9 and 1.4–2.5 log CFU/g in cheese and brines, respectively. However, the addition of *L. reuteri* and glycerol to water used in the preparation of brines reduced *L. monocytogenes* by 1.2–2.6 and 1.2–2.2 log CFU/g in cheese and brines, respectively. The highest inhibitory effect of *L. reuteri* was observed against *L. monocytogenes* in both cheese and brine with 10% NaCl and high temperatures (10–24 °C). The addition of *L. reuteri* with glycerol has the potential to reduce the risk of *L. monocytogenes* without negatively affecting the physicochemical characteristics of the cheese.

## 1. Introduction

White-brined cheese (WBC) is a common type of cheese in different Eastern Mediterranean countries and neighboring regions. WBC is a typically fresh unripened, rennet-coagulated cheese, with a soft to semi-hard texture [1]. It is considered a ready-to-eat (RTE) product that can be consumed fresh or after storage in a brine solution containing a high salt content (10–24% NaCl), most often kept refrigerated [2]. However, sometimes WBC is stored at room temperature [3], and produced seasonally from goat or ewe milk, or their combination, in un-mechanized conditions at various phases of production [4]. WBC could be contaminated at any stage of production with different foodborne pathogens, including *Escherichia coli* O157:H7, *Salmonella* spp., *Staphylococcus aureus*, and *Listeria monocytogenes* [5,6].

*L. monocytogenes* is a Gram-positive bacterium that is recognized as a ubiquitous pathogen and a causative agent of foodborne listeriosis, with a high mortality rate of nearly 20–30% [7], particularly in high-risk groups including pregnant women, infants, elderly, immunocompromised people, and, on rare occasions, people without any underlying conditions [8]. This pathogen is a cause of concern for different types of industries. In fact, *Listeria* spp. are widely spread in the environment [9] and may also colonize foods and food contact surfaces, such as polystyrene, stainless steel, and glass, at any point during food processing [10,11]. *Listeria* spp., mainly *L. monocytogenes*, have a remarkable tolerance to relatively extreme pH (can survive but not grow from 3.3 to 4.2), refrigerated temperatures (between 4 and 10 °C), and high salt concentrations (from 0.5 to 12%), and can form biofilms on surfaces waste water pipes, bends in pipes and rubber seals, as well as improperly sanitized equipment [12]. Consequently, these characteristics make *L. monocytogenes* a perilous hazard for a wide range of food products, including dairy products, particularly cheeses, which are highly perishable with a limited shelf life even after applying different preservative hurdles such as brine solution and refrigeration [13]. Since 1985, many listeriosis outbreaks linked to different types of cheese have been reported [14]. In addition, the presence of *L. monocytogenes* in cheeses causes a significant economic burden to the dairy industry in terms of product testing, product loss, and recalls [15], which requires particular attention to use different control strategies, including physical treatments such as high-pressure and irradiation, to enhance the microbial safety of cheese by controlling the growth and survival of foodborne pathogens, including *L. monocytogenes*, during processing and storage [16]. Although these techniques were considered effective in preventing the growth of foodborne pathogens in food, in the meantime, they also showed limitations in their application, such as the need for high capital investment or affecting the quality parameters of foods [12]. Therefore, there is a culminating interest in naturally occurring and safe antimicrobial strategies such as bio-preservatives in food processing and preservation, which have the potential to improve the overall quality and safety of food products [17].

The use of bacteriocins or bacteriocin-producing lactic acid bacteria (LAB) is a promising technique to enhance cheese safety by preventing the growth of foodborne pathogens, including *L. monocytogenes* [18]. Bacteriocins are generally recognized as safe (GRAS) for consumers, since they can be degraded by proteases in the gastrointestinal tract (GIT) [19]. These compounds can be incorporated in different forms, including direct addition to the food, immobilized form on the packaging, or inoculation of foods by bacteriocin-producing LAB [18]. Several studies have established that LAB promote microbial food safety based on various synergistic mechanisms. LAB synthesize a variety of natural antimicrobial compounds, e.g., organic acids (e.g., lactic, acetic, and propionic acids), that lower the environmental pH and compromise the structural integrity of microbial membranes. Bacteriocins such as nisin and plantaricins function by pore formation or inhibition of cell wall synthesis in target pathogenic bacteria. LAB also contend with the pathogens for the required nutrients and adhesion sites, thereby inhibiting their colonization and growth. There exist a few limitations, however, like strain dependency, variable efficacy on food matrix and storage conditions, and potential sensory effects. In addition, while bacteriocins have excellent potential as antimicrobials, their spectrum of activity may be narrow, and few compounds (e.g., nisin) are actually cleared for use in foods. These aspects require further research in order to optimize delivery systems and improve stability and effectiveness of LAB-based bio-preservation strategies [20,21,22].

*Lactobacillus reuteri* is an obligatory heterofermentative bacterium [23], often found in probiotic and fermented foods, including cheeses. It has an antimicrobial activity against molds, yeasts, parasites, and a wide range of pathogenic bacteria (including *L. monocytogenes*) due to the production of reuterin [2,24]. Reuterin is a water-soluble aldehyde with a high potential to be used as a food preservative due to its resistance to proteolytic and lipolytic enzymes and its ability to be active under a broad range of pH conditions [25]. Therefore, this study aimed to investigate the effect of *L. reuteri* as a bio-preservative combined with different salt concentrations on the inhibition of *L. monocytogenes* in WBC at different storage temperatures. Additionally, the effect of *L*. *reuteri* inoculation method (through pasteurized milk, distilled water, or brine solution) was investigated.

## 2. Materials and Methods

### 2.1. L. monocytogenes Strains and Culture Preparation

Five *L. monocytogenes* strains (Lis-2-138, Lis-2-243, GLM-1, GLM-3, and GLM-5) isolated from meat or dairy product plants, obtained from the culture collection of the Food Microbiology Laboratory at the Hashemite University, were used in this study. The activation of bacterial cultures was done by taking one loopful from the frozen cultures of each *L. monocytogenes* strain and streaking onto the surface of Tryptone Soy Agar (TSA, Oxoid, Basingstoke, UK) plates, incubating at 37 °C for 24 h, then a single colony from each *L. monocytogenes* strain was streaked on *Listeria* Selective Agar Base with *Listeria* Selective Supplement (LSA, Oxoid, Basingstoke, UK) and incubated aerobically at 37 °C for 48 h. Thereafter, a single colony of each *L. monocytogenes* strain was transferred into 10 mL of Brain Heart Infusion (BHI) broth (Oxoid Ltd., Basingstoke, UK) and incubated at 37 °C for 24 h. Thereafter, 0.1 mL of this culture was transferred to fresh BHI broth and incubated aerobically overnight at 37 °C. For experimental use, another final culture transfer was carried out in BHI broth.

### 2.2. L. reuteri Strains and Culture Preparation

Five *L. reuteri* strains (SS730, S3608, CF2, MM-2, and RC14) originally provided by the Department of Nutrition and Food Sciences, University of Manitoba, Canada, were used in this study. A loopful of each frozen culture was streaked onto MRS agar (Oxoid Ltd., Basingstoke, UK) and incubated at 37 °C for 24 h under anaerobic conditions using a CO_2_ incubator (Astec Co., Ltd., Fukui, Japan) with O_2_ less than 5% and 95% N_2_. A single colony of each *L. reuteri* strain was transferred into MRS broth for activation before experimental use. Then, *L. reuteri* strains were sub-cultured twice in MRS broth (Oxoid Ltd., Basingstoke, UK) anaerobically for 24 h at 37 °C.

### 2.3. Cocktail Preparation of L. monocytogenes and L. reuteri

To prepare the 10 mL bacterial cocktails, 2.0 mL of each strain of *L. monocytogenes* or *L. reuteri* was transferred into sterile empty tubes. The cocktails of *L. monocytogenes* or *L. reuteri* were separately mixed thoroughly and centrifuged at 3000× *g* for 18 min (Nüve, Istanbul, Turkey), and then the supernatants were discarded and 10 mL of 0.1% peptone water (Oxoid Ltd., Basingstoke, UK) was added to wash the pellets of each cocktail using a vortex mixer. The cultures were centrifuged and washed again, and the pellets of *L. monocytogenes* were finally collected in 10 mL of 0.1% peptone water to yield 8–9 log CFU/mL; the pellets of *L. reuteri* were collected in 1 mL of 0.1% peptone water to yield 9–10 log CFU/mL.

### 2.4. Cheese Production

The WBC production was prepared according to Al-Nabulsi et al. [2] at the dairy factory at the Jordan University of Science and Technology, Jordan. Each treatment was prepared using whole-fat raw bovine milk (15 L) obtained from a local supplier. The milk was pasteurized at 72 °C for 15 s and cooled to 36–37 °C. The pasteurized milk was screened for the presence of *L. monocytogenes* by taking 6 pasteurized milk samples plated on LSA (Oxoid, Basingstoke, UK) and incubated for 48 h at 37 °C. Afterward, the pasteurized milk was coagulated with diluted single-strength calf rennet (Dairy Connection, Inc., Madison, WI, USA) in sterile distilled water (1:10) and added to the milk for 30–40 min, to yield a milky texture that was cut with a knife to allow releasing whey. The curds were transferred in a sterilized cheesecloth to a perforated sterile steel mold (50 × 50 × 2 cm in length, width, and height, respectively) and pressed with a stainless-steel plate for 30 min. The cheese was manually cut with a sterile knife into 20–25 g pieces (5 × 5 × 2 cm in length, width, and height, respectively) that were immersed in either 10 or 15% (*w*/*v*) NaCl brine solutions in a ratio of 1:4 (cheese:brine).

**Figure 1:**
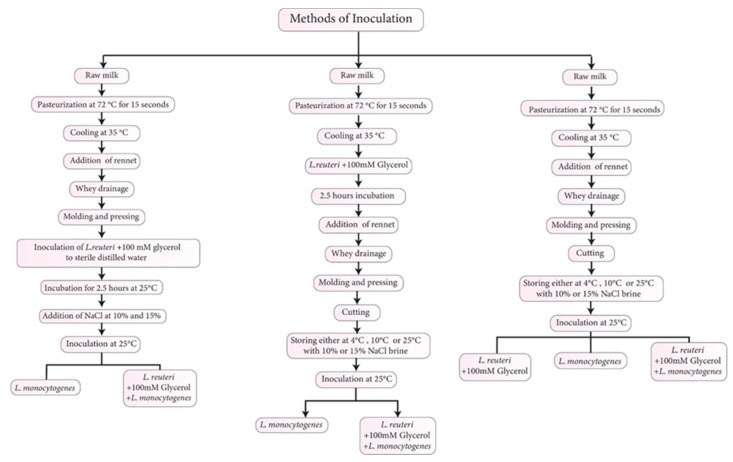
Inoculation of *L. reuteri* and *L. monocytogenes* during different stages of white-brined cheese processing.

The *L. monocytogenes* cocktail was inoculated into the brine solution to yield ~5.0 log CFU/g. The *L. reuteri* cocktail was inoculated with 100 mM glycerol (Panreac Química, Barcelona, Spain) into the WBC to yield ~6.0 log CFU/g at three different steps during the processing, including direct inoculation to the brine solution, to the distilled water used to prepare the brine solution and held for 2.5 h before adding the salt, or to the pasteurized milk that was held for 2.5 h before adding the rennet enzyme (Figure 1). Then, the cheese samples were stored at 4, 10, and 24 °C for 91 days (Table 1).

### 2.5. Sampling and Microbiological Analysis

The viability of *L. monocytogenes* and *L. reuteri* in the cheese samples and brine solution was analyzed at specific intervals (0, 3, 7, 14, 21, 28, 49, 71, 91 days). A cross-sectioned 5 g piece of WBC sample was taken by using a sterile spoon, or 5 mL of brine was taken via micropipette under aseptic conditions and homogenized with 45 mL of 0.1% peptone water (Oxoid Ltd., Basingstoke, UK) by shaking for 2 min in a sterile stomacher bag using a stomacher (Easy Mix, AES Laboratories, Combourg, France).

A proper dilution of stomached cheese or brine samples was prepared by diluting the samples in 0.1% peptone water, and then 100 μL was plated on the surface of LSA (Oxoid, Basingstoke, UK) and incubated for 48 h at 37 °C for enumeration of *L. monocytogenes,* and Rogosa agar (Oxoid Ltd., Basingstoke, UK) for enumeration of *L. reuteri*, and incubated under anaerobic conditions in a CO_2_ incubator (ASTEC Ltd., Suita, Osaka, Japan) for 48 h at 37 °C.

### 2.6. Chemical Analysis

#### 2.6.1. Water Activity (a_w_) Measurements

The a_w_ of WBC at different day intervals was assessed using an a_w_ meter (Novasina AG, Labmasters AW, Lachen, Switzerland) by analyzing ca. 2 g from a cross-section of a WBC sample at 21 °C for all of the day intervals at 4, 10, and 24 °C in 10% or 15% brine solutions.

#### 2.6.2. Salt Determination

The NaCl concentration in WBC samples was determined by the AOAC method [26]. About 1.0–1.5 g of the cheese samples at different day intervals was taken and held in a muffle furnace (Barnstead Thermolyne, IA, USA) for 8 h at 550 °C. The ashed sample was mixed with 25 mL of distilled water. Then, the mixture was titrated using 0.05 N AgNO_3_ (Carlo Erba, Val de Reuil, France) after adding drops (0.5 mL) of 0.5 N potassium chromate (Alpha Chemika, Mumbai, India) as an indicator. The salt concentration was calculated by applying the following equation:

%Salt content = (titrated volume of AgNO_3_ mL × 0.00292) ÷ mass of sample (g) × 100.

#### 2.6.3. pH Measurements

The pH values of the WBC and brine solution samples at the corresponding time intervals were determined using a pH meter (Adwa pH meter, AD 1000, Adwa, Nușfalău, Romania).

### 2.7. Statistical Analysis

Statistical analysis was carried out using the Statistical Package for Science (SPSS) software version 22.0 (2013, IBM Corp., Armonk, NY, USA). Each reported value was the mean of two experiments for each measurement, and the results were expressed as means ± SD. The data obtained were subject to analysis of variance (ANOVA) to test the effects of different treatment factors (absence or presence of *L. monocytogenes* and *L. reuteri*, brine concentration, and storage time and temperature), and Tukey’s HSD test was used to determine the statistical significance of the effects at a *p*-value of 0.05 among treatments or storage time.

## 3. Results and Discussion

### 3.1. pH of WBC and Cheese Brine

Table 2 and Table 3 show the pH values of the WBC and cheese brines, respectively, in the presence of *L. monocytogenes* only or with *L. reuteri* stored at 4, 10, and 24 °C for 91 d. The initial pH values of WBC samples in the presence of *L. monocytogenes* only or with *L. reuteri* stored in 10% and 15% NaCl solution ranged between 6.3 and 6.5 at day 0 and changed to 5.7–6.4, 5.2–6.3, and 4.9–5.7 after 91 d of storage at 4, 10, and 24 °C, respectively, regardless of the *L. reuteri* inoculation method (Table 2). The initial pH values of cheese brine samples of 10% and 15% NaCl in the presence of *L. monocytogenes* only or with *L. reuteri* ranged between 6.4 and 6.8 at day 0 and decreased to 5.7–6.4, 5.5–6.2, and 4.9–6.6, respectively, after 91 d of storage at 4, 10, and 24 °C, respectively, regardless of the *L. reuteri* inoculation method (Table 3).

The highest reductions in pH values of cheese or brine were observed at the highest storage temperature (24 °C) at d 91 d, regardless of salt concentration and the presence of *L. monocytogenes*, *L. reuteri*, or both. This finding aligns with a study that analyzed the pH values of cheese samples stored under various storage conditions and found that the pH value declined from 5.8 to 5.2 after 29 d of storage at 5 °C, while the pH value at 24 °C decreased to 4.8 at day 14 [27]. Another study also indicated that pH value of soft white cheese significantly declined from 4.8 to 4.7 after 100 d of storage at 8 °C [28]. Al-Nabulsi et al. [2] indicated that lower pH values of WBC and brine samples were found in 10% brine compared to the 15% brine solution, where the pH values ranged from 4.7 to 6.1 and from 4.6 to 6.0, respectively, in 10% brine compared to 5.2–6.1 and 5.0–6.2, respectively, in 15% brine in the presence of *E. coli* O157:H7, *L. reuteri*, or both. In the current study, there were fluctuations in the pH values of cheese and brine at different salt concentrations, although it is expected that the numbers of LAB, proteolytic, and lipolytic activities during ripening and microbial lactose metabolism decrease as the level of NaCl increase [2,3]. Furthermore, the pH reduction may be due to the production of organic acids by inoculated and naturally occurring LAB [29].

### 3.2. Water Activity of White-Brined Cheese

Table 4 shows the a_w_ values of all cheese samples inoculated with *L. reuteri*, *L. monocytogenes*, or both and stored in a 10% or 15% brine solution at 4, 10, or 24 °C. The initial a_w_ values of cheese were 0.92–0.96 in a 10% or 15% brine solution. However, the final a_w_ values of cheese samples remained constant and were not affected by storage temperature, NaCl concentration, or presence of *L. monocytogenes*, *L. reuteri*, or both after 91 d of storage, with a range of 0.90–0.95 (Table 4). Similar results were obtained by Al-Nabulsi et al. [2], who reported that the a_w_ values of cheese stored in 10% or 15% brine solutions were 0.90–0.95 after 28 d of storage at 10 and 24 °C. Osaili et al. [5] also indicated that the a_w_ in cheese stored in 15% brine at 10 or 21 °C ranged from 0.94 to 0.95 from day 1 to 0.93 to 0.94 at day 28. However, the a_w_ of cheese stored in 15% brine was 0.92 on day 1 at both temperatures and decreased to 0.88–0.90 after 28 d of storage.

### 3.3. Salt Content of WBC

The salt concentration is a significant factor that affects the shape, texture, flavor, and quality of cheese [26]. Temperature and storage time significantly impacted the salt content of cheese [30]. In the current study, the salt contents of WBC samples were 4.8–6.1, 5.6–5.7, and 4.6–5.5% at 4, 10, and 24 °C, respectively, after 12 h of immersion in a 10% brine solution, and increased to reach 6.3–6.8, 6.3–6.9, and 6.0–6.7%, respectively, after 91 d of storage. While the salt contents of cheeses stored in 15% brine solution were 4.3–5.4, 4.9–5.2, and 4.8–5.2 at 4, 10, and 24 °C, respectively, after 12 h of immersion in the brine solution and increased to reach 6.6–6.8, 6.6–6.8, and 6.5–7.3%, respectively, after 91 d of storage (Table 5). This increase is due to the diffusion process that occurs during storage, where NaCl molecules move from the brine into the cheese and water diffuses out through the matrix, which increases the salt content and reduces the a_w_ [2].

It was evident that the salt content of WBC increased as the brine solution and temperature increased, with higher levels of salt observed in cheeses stored in a 15% brine solution at 10 and 24 °C. Similarly, Setyawardani et al. [30] reported that the refrigerated artisanal goat cheese samples had a higher salt content than the frozen cheeses, and the salt content increased as the storage time increased. Kaya [31] also reported that increasing salt content in the brine has a substantial effect on the salt contents of Gaziantep cheese, where higher brine concentrations resulted in greater salt uptake by the cheese during storage. Moreover, Al- Nabulsi et al. [2] pointed out that the salt content of WBC stored in 15% brine at 4 and 25 °C was higher than samples stored in 10% brine solution. The salt levels in cheese stored in 15% brine solution were 5.3–7.7% after 28 d, while the salt content of cheese stored in a 10% brine solution was 4.5–6.7% after 28 d.

### 3.4. Behavior of L. reuteri in White-Brined Cheese Inoculated at Different Stages of Processing in the Presence of L. monocytogenes at Different Storage Temperatures 

The behavior of *L. reuteri* in cheese stored in 10% or 15% NaCl brine inoculated with *L. monocytogenes* at 4, 10, or 24 °C for 91 d was investigated. The initial numbers of *L. reuteri* (6.1–6.5 log CFU/g) significantly (*p* < 0.05) increased to 7.3, 6.7, and 6.6 log CFU/g in cheese made with *L. monocytogenes* after 91 d of storage in 10% NaCl at 4, 10, and 24 °C, respectively (Table 6). The growth of *L. reuteri* in cheese at 4 or 10 °C is explained by the production of cold shock proteins that enable microorganisms to overcome the environmental stresses. In another study, Chen et al. [32] found that *L. reuteri* survived and remained at steady levels in drinkable yogurt, with numbers of approximately 4.0 log CFU/g in thin yogurt stored at 37 °C for 4 weeks and 5.0 log CFU/g in thick yogurt stored at 28 °C for 5 months.

Meanwhile, the initial *L. reuteri* numbers (6.1–6.7 log CFU/g) decreased in WBC stored in 15% NaCl and reached to 5.3–5.5 log CFU/g at 4, 10, and 24 °C by the end of the storage period. This inhibitory effect could be attributed to the high salt content, which may have led to osmotic stress [33]. Moreover, Al-Nabulsi et al. [2] reported that for *L. reuteri*, the initial numbers were significantly reduced in cheese made without *E. coli* O157:H7 in 10% brine at 10 °C, from 6.2 to 5.1 log CFU/g after 28 d. In 10% brine at 25 °C and in 15% NaCl at 10 °C, the initial numbers of *L. reuteri* in cheese made without *E. coli* O157:H7 decreased from 5.9 to 5.1 log CFU/g after 28 d.

It was evident that *L. reuteri* inoculated into WBC at different preparation stages was not affected by the presence of *L. monocytogenes.* In contrast, Langa et al. [34] indicated that *L. reuteri* decreased by ~1.5 log CFU/g when co-cultured with *E. coli* O157:H7 and *L. monocytogenes* in semi-hard cheese stored at 12 °C for 30 d and found that the survival of *L. reuteri* plus glycerol decreased from 6.8 at day 1 to 2.6 after 30 d of storage. Al-Nabulsi et al. [2] reported that the numbers of *L. reuteri* increased from 6.2 to 8.3 and 7.8 log CFU/g in WBC made with *E. coli* O157:H7 and stored in 10% or 15% brine, respectively, at 25 °C after 28 d; in 15% brine at 10 °C, the numbers of *L. reuteri* in WBC made with *E. coli* O157:H7 significantly decreased from 6.2 to 5.2 log CFU/g.

### 3.5. Behavior  of L. reuteri in Cheese Brine Inoculated at Different Stages of Processing in the Presence of L. monocytogenes at Different Storage Temperatures

The behavior of *L. reuteri* in cheese brine at 10% or 15% NaCl inoculated with *L. monocytogenes* and stored at 4, 10, or 24 °C for 91 d was investigated (Table 7). The initial number of *L. reuteri* (5.5–6.4 log CFU/g) slightly increased in 10% cheese brine stored at 4 (7.1 log CFU/g), 10 °C (7.3 log CFU/g), and 24 °C (7.5 log CFU/g). However, the numbers changed to 5.6–6.4 log CFU/g in a 15% brine solution at different storage temperatures. On the other hand, the inoculation of *L. reuteri* into milk used in cheese processing or distilled water used in brine preparation with glycerol in the presence of *L. monocytogenes* showed lower numbers at the end of the storage period, and reached 6.2–6.8 log CFU/g in 10% brine and 5.4–5.6 log CFU/g in 15% brine.

Similar results were obtained by Al-Nabulsi et al. [2], who reported that *L. reuteri* numbers significantly increased from 5.0 to 5.3 log CFU/mL at d 1 to reach 5.7 to 7.8 log CFU/mL after 28 d in the presence of *E. coli* O157:H7 in 10% or 15% NaCl brine concentrations at 25 °C. The increased presence of *L. reuteri* could be due to the cell surface proteins, as reported by Singh et al. [35], who reported that different strains of *L. reuteri* were resistant to the highly acidic environment compared to the strains treated in 5 M LiCl for the removal of cell surface proteins, which are a protective sheath against hostile environmental agents such as acidic and high-NaCl environments. A study by Rasch [36] reported that the different environmental parameters, including pH (4.5–6.5) and NaCl (0.5–3%), did not interfere with the efficiency of reuterin against *E. coli* K12.

### 3.6. Inhibitory  Effect of L. reuteri Inoculated at Different Stages of White-Brined Cheese Processing Against L. monocytogenes in Cheese at Different Storage Temperatures

WBC has high nutrient content, high water activity, and high buffering capacity; therefore, it supports the growth of spoilage and foodborne pathogens, such as *L. monocytogenes* [37]. This was confirmed by the findings of this study, which investigated the behavior of *L. monocytogenes* in WBC made with or without *L. reuteri* and stored in 10% or 15% NaCl at 4, 10, or 24 °C for 91 d (Table 8). The initial numbers of *L. monocytogenes* (4.6–4.8 log CFU/g) in cheese made without *L. reuteri* at 4, 10, and 24 °C significantly increased (*p* < 0.05) during the 91 d storage time and reached to 6.9, 6.4, and 6.7 log CFU/g, respectively, in cheese stored with 10% brine and to 6.2, 6.3, and 6.0 log CFU/g, respectively, in cheese stored with 15% NaCl. Although factors including temperature, pH, and salt content affect the growth of *L. monocytogenes* in cheese, the microbe still has the ability to adapt and grow in the presence of highly osmotic environments [38]. In the current study, the salt contents of WBC samples stored in 10% and 15% brine solutions reached 6.0–6.9% and 6.5–7.3%, respectively, by the end of the 91 d storage period (Table 5). Further, the initial pH values of cheese were 6.3–6.5 and reduced to 4.9–6.4 (Table 2), with a final a_w_ of 0.9–0.95 (Table 4) after 91 d at different storage temperatures.

It has been reported that when *L. monocytogenes* was exposed to sub-lethal environmental conditions, it may provide cross-protection against other factors such as salt, acid, alkaline pH, ethanol, heat, and oxidative stress [39]. For example, a study by Ilhak et al. [40] indicated that the survivability of *L. monocytogenes* in Turkish white cheese prepared from raw milk that was ripened in high-salt brine (6.7%) for 15 d at 4 °C was improved when the pathogen was pre-exposed to a sub-lethal acidic environment (pH 4.6–4.8). Faleiro et al. [41] noted that foods with a low pH (5.0), such as cheese, may induce an acid tolerance response in *L. monocytogenes.* Additionally, Cataldo et al. [42] indicated that acid-adapted *L. monocytogenes* survived significantly better compared to un-adapted cells in Crescenza cheese, a soft cheese with 4–10% NaCl and pH 5.0–5.6, after 14 d of storage at 4 °C. Additionally, Kapetanakou et al. [43] reported that *L. monocytogenes* persisted at the levels > of 2 log CFU/g in Cottage cheese with pH 5.0 during the entire product shelf life.

Numerous studies have demonstrated that some bacteria, including *L. monocytogenes*, adapt to high osmolality by synthesizing or uptaking suitable solutes such as proline and carnitine to maintain equilibrium between the intracellular and extracellular environments [44]. These solutes are extremely soluble substances, known as protective osmolytes, that play a significant role in enabling the cells to restore their osmotic balance without negatively influencing the cell structure or function [45]. Likewise, *L. monocytogenes* survived in different types of cheese, such as curd and soft cheeses, for long periods at refrigeration and room temperature, with high survival rates at lower temperatures [46]. It was also reported that *L. monocytogenes* was more resistant to salt in cheese at low temperatures (8 °C) [41].

Salting is widely used in the food industry for food preservation as it reduces a_w_, thus allowing a considerable increase in the storage time of food products. It is worth mentioning that the levels of salt, pH, and a_w_ used in the current study did not prevent the growth of *L. monocytogenes* at 4, 10, and 24 °C. Furthermore, typical cheese preservation procedures may not only fail to prevent the growth or survival of *L. monocytogenes*, but may markedly improve their virulence [47]. However, the addition of other factors, such as bacteriocins or LAB cultures producing bacteriocins, to WBC may substantially reduce the numbers of *L. monocytogenes* in combination with other factors such as salt and low pH. On the other hand, the addition of *L. reuteri* to WBC at different stages of processing significantly (*p* ˂ 0.05) affected the survivability of *L. monocytogenes* at different storage temperatures. At the end of the 91 d storage time, the *L. monocytogenes* count for the control (cheese un-inoculated with *L. reuteri*), regardless of the brine concentration or the storage temperature, was 6.0–6.9 log CFU/g. However, *L. monocytogenes* counts decreased significantly (*p* ˂ 0.05) to 4.9–5.9 log CFU/g when *L. reuteri* was added with glycerol to the cheese brine, to 4.1–5.0 log CFU/g when *L. reuteri* was added with glycerol to the distilled water used to prepare cheese brine, or to 3.5–5.0 log CFU/g when *L. reuteri* was added to the pasteurized milk during processing (Table 6). It is worth mentioning that the addition of *L. reuteri* to pasteurized milk showed the most inhibitory effect against *L. monocytogenes* in cheese stored in 10% NaCl at 10 °C. Further, the addition of *L. reuteri* to pasteurized milk during processing or sterile water used for brine preparation was more effective against *L. monocytogenes* in cheese compared to its inoculation in cheese brine.

The decrease in *L monocytogenes* counts could be attributed to the inhibitory effects of *L. reuteri* and its bacteriocin, reuterin. Similar results were obtained by Al-Nabulsi et al. [2], who reported that an *L. reuteri* cocktail reduced the numbers of *E. coli* O157:H7 in WBC by 2.6 log CFU/g after 28 d of storage in 10% NaCl brine at 25 °C. In the current study, it is evident that *L. reuteri* has a significant role in the reduction of *L. monocytogenes* due to its capability to produce antimicrobial molecules including organic acids, inhibitory enzymes, hydrogen peroxide, and reuterin [48].

Similarly, Langa et al. [34] reported that *L. reuteri* INIA P572 generated reuterin in the presence of 100 mM glycerol during semi-hard cheese production and ripening to quantities that showed a bactericidal effect against *L. monocytogenes*, which was not detected after 7 d of storage. Furthermore, *L. monocytogenes* and *E. coli* O157:H7 were not detected in cheese treated with 5.30 mM/g of reuterin by 7 d at 12 °C [34].

The current study is further proof that *L. reuteri* has a potent antibacterial activity against *L. monocytogenes* in both 10 and 15% brine at different storage temperatures (4, 10, and 24 °C). This could be related to the quorum-sensing mechanism of *L. reuteri*, which promotes reuterin production during growth [49]. Reuterin is known to be an analog of D-ribose, which inhibits ribonucleotide reductases, and are necessary for the de novo synthesis of deoxyribonucleotides needed for DNA synthesis [50]. This could explain the considerable reduction in *L. monocytogenes* populations in cheeses made with *L. reuteri* with glycerol after 91 days at different levels of brine and storage temperatures. *L. reuteri* survival increased with decreasing salt concentrations and increasing temperature (Table 6 and Table 7), which may enhance production and produce an even higher quantity of reuterin compared to when using high brine concentrations, which may cause bacterial cell dehydration and osmotic stress and decrease the reuterin yield [32].

Apparently, reuterin and salt showed synergistic effects against *L. monocytogenes*. Similarly, previous studies showed that increasing salt content enhanced the antimicrobial activity of reuterin against *L. monocytogenes* and *E. coli* O157:H7 [50]. The addition of 3-HPA at 2 AU/mL with nisin (100 IU/mL) and lactoperoxidase system (0.2 ABTSU/mL) synergistically inactivated *L*. *monocytogenes* and *Staphylococcus aureus* in Cuajada cheese (curdled milk) after 12 d of storage at 10 °C [51]. In another study, *E. coli* O157:H7, *Salmonella enteritidis*, and *L. monocytogenes* were inhibited in acidified milk at pH 5.0 by adding 1 AU/mL 3-HPA with 100 mg/kg diacetyl [52]. Furthermore, the application of high-pressure processing at 450 MPa/5 min with the addition of 16 mM of 3-HPA reduced the numbers of *L. monocytogenes* in cooked ham by 1.7 and 2.6 log CFU/g at 4 and 10 °C, respectively, by 35 d of storage and reduced *S. enterica* numbers by 2 and 1 log CFU/g at 4 and 10 °C, respectively, compared to control samples [53]. Reuterin treatment of cold-smoked salmon at 10 AU/g effectively reduced *L. monocytogenes* by 2.0 log CFU/g by 15 d of storage at 8 °C or by 1.4 log CFU/g by day 1 at 30 °C, compared to control samples [54]. In another study, the addition of reuterin to creamed cottage cheese at 50 AU/g decreased *L. monocytogenes* numbers by 1.5 log CFU/g by 21 d of storage at 7 °C [55].

### 3.7. Inhibitory  Effect of L. reuteri Inoculated at Different Stages of White-Brined Cheese Processing Against L. monocytogenes in Cheese Brine at Different Storage Temperatures

The brine could be a possible cause of contamination in cheeses via transferring foodborne pathogens such as *L. monocytogenes* from brine to cheese. Commercial cheese brines are frequently used in WBC, generating a brine that is rich in nutrients that are released from cheese and perhaps encouraging the growth of pathogenic microorganisms [3]. This may explain the capability of *L. monocytogenes* to survive and grow in 10% or 15% brine of cheese made without or with the addition of *L. reuteri* at 4, 10, or 24 °C (Table 9).

It was clear that the minimum growth rate of the *L. monocytogenes* population was at the higher salt concentration at all storage temperatures. The number of *L. monocytogenes* at day 0 was 5.5–5.8 log CFU/mL, and the relatively low number could be due to the high salt concentration and absence of protective matrix in the brine, such as fat and protein [5].

*L. monocytogenes* numbers in both 10% and 15% brine un-inoculated with *L. reuteri* at 4, 10, or 24 °C significantly (*p* ˂ 0.05) increased to 6.1–6.8 log CFU/mL by the end of 91 d storage period (Table 7). Barancelli et al. [56] reported that *L. monocytogenes* was isolated from brine samples from different cheese factories. Further, Boyer et al. [57] reported that no significant decline in *L. monocytogenes* numbers was obtained in chill brines with salt levels of 7.9–13.2% after 10 d of storage at 4 and 12 °C.

However, the initial *L. monocytogenes* number (5.3–5.8 log CFU/mL) decreased to 4.4–5.9, 3.6–4.6, and 4.9–6.4 log CFU/mL in 10% NaCl brine at 4, 10, and 24 °C, respectively, and to 4.1–5.4, 4.2–5.5, and 5.1–5.5 log CFU/mL in 15% NaCl brine at 4, 10, and 24 °C, respectively, when *L. reuteri* and glycerol were added at different stages of cheese processing by 91 d of storage. It was evident that adding *L. reuteri* to brine was the least effective *L. reuteri* delivery method against *L. monocytogenes* in cheese brine, and this could be due to the inhibitory effect of salt against *L. reuteri* and the decrease in reuterin yield. On the other hand, inoculation of *L. reuteri* into the milk used in the processing of WBC yielded the most inhibitory effect against *L. monocytogenes* in cheese brine, and this could be due to the presence of nutrients that support the growth of *L. reuteri* and enhanced production of reuterin. Al- Nabulsi et al. [2] reported that the numbers of *E. coli* O157:H7 (6.9 log CFU/mL) in 15% NaCl brine containing *L. reuteri* was reduced to 4.4 CFU/mL after 28 d of storage of WBC. Langa et al. [52] reported that adding reuterin (1 AU/mL) with Diacetyl (100 mg/kg) in acidic milk (pH = 5) was effective in decreasing *L. monocytogenes* from an initial level of ca. 6.0 log CFU/mL to 3.6 log CFU/g after 24 h; with normal pH milk (pH = 6.8), the count increased to ca. 6.9 log CFU/mL.

## 4. Conclusions

Using *L. reuteri* as an adjunct culture to cheese may pose a considerable antimicrobial activity against *L. monocytogenes*. This is potentially due to the production of antimicrobial substances such as reuterin. The findings of this study confirmed the protective effect of reuterin-producing *L. reuteri* with 100 mM glycerol in the production of cheese contaminated with *L. monocytogenes*. *L. monocytogenes* was capable of surviving in WBC (immersed into 10 or 15% brine concentrations) at different storage temperatures (4, 10, or 24 °C for 91 d). In the absence of *L. reuteri*, numbers of *L. monocytogenes* increased by ca. 2 logs after 3 d of storage; this increase persisted till the end of the 91 d of storage at the different storage temperatures, and the growth was more apparent in the WBC immersed into the 10% brine compared to the 15% brine solutions. Using glycerol (100 mM) in the different *L. reuteri* delivery methods significantly decreased the numbers of *L. monocytogenes* in both cheese and brine solutions compared to the control. Delivering *L. reuteri* along with glycerol to pasteurized milk used in cheese processing was the most effective inoculation method in inhibiting the growth of *L. monocytogenes* in BWC compared to inoculating *L. reuteri* into distilled water or brine solution. *L. reuteri* was able to grow in cheese immersed into 10 or 15% brine solutions under the different storage temperatures. Yet, *L. reuteri* growth was more prominent when using 10% brine solution at 24 °C. These conditions may enhance the production of reuterin and other metabolites by *L. reuteri*, which may entail higher inhibitory activity against *L. monocytogenes* in cheese.

The future prospects for *L. reuteri* and glycerol in food products are positive, with chances to improve food safety, increase shelf life, provide functional advantages, and match customer demands for natural ingredients. However, realizing this potential will require further research, such as investigating the antagonistic of *L. reuteri* against other foodborne pathogens in WBC and investigating the antagonistic of *L. reuteri* against *L. monocytogenes* and other foodborne pathogens in other fermented products. Further, the allied combinations of *L. reuteri* and glycerol with other antibacterial agents or food preservatives like essential oils or organic acids could be investigated.

## Figures and Tables

**Table 1:** The experimental design used for survival studies of *Listeria monocytogenes* in white-brined cheese and brine solution.

Treatments	*L. monocytogenes* ^1^	*L. reuteri* ^2^	Percentage of NaCl in Brine	Storage Temperature
T1	+	−	10%	4 °C
T2 *	+	+	10%	4 °C
T3 **	+	+	10%	4 °C
T4 ***	+	+	10%	4 °C
T5	+	−	15%	4 °C
T6 *	+	+	15%	4 °C
T7 **	+	+	15%	4 °C
T8 ***	+	+	15%	4 °C
T9	+	−	10%	10 °C
T10 *	+	+	10%	10 °C
T11 **	+	+	10%	10 °C
T12 ***	+	+	10%	10 °C
T13	+	−	15%	10 °C
T14 *	+	+	15%	10 °C
T15 **	+	+	15%	10 °C
T16 ***	+	+	15%	10 °C
T17	+	−	10%	24 °C
T18 *	+	+	10%	24 °C
T19 **	+	+	10%	24 °C
T20 ***	+	+	10%	24 °C
T21	+	−	15%	24 °C
T22 *	+	+	15%	24 °C
T23 **	+	+	15%	24 °C
T24 ***	+	+	15%	24 °C

(+): The microbe was inoculated; (−): the microbe was not inoculated. * Adding the *L. reuteri* and 100 mM glycerol directly to 10% or 15% brine solution. ** Adding the *L. reuteri* and 100 mM glycerol to the sterile distilled water that was used to prepare the 10% or 15% brine solution. *** Adding the *L. reuteri* to pasteurized milk that was used to prepare the WBC. ^1^ The final count of *L. monocytogenes* in WBC was ~5.0 log CFU/g. ^2^ The final count of *L. reuteri* in WBC was ~6.0 log CFU/g.

**Table 2:** pH values ^1^ of white-brined cheese inoculated with *L. monocytogenes* or *L. reuteri* and *L. monocytogenes* and stored in 10% or 15% brine solution at 4 °C, 10 °C, and 24 °C for 91 days.

Storage Time (Days)									
Treatments ^2^	0 ^3^	3	7	14	21	28	49	71	91
T1	6.40 ± 0.02 ^Ae^	6.30 ± 0.02 ^CDEFGcde^	6.38 ± 0.04 ^DEFGHde^	6.27 ± 0.00 ^FGcd^	6.21 ± 0.01 ^FGbc^	6.30 ± 0.04 ^FGcde^	6.12 ± 0.03 ^GHb^	5.73 ± 0.06 ^Da^	5.68 ± 0.00 ^DEa^
T2	6.35 ± 0.00 ^Ac^	6.35 ± 0.03 ^DEFGHIJc^	6.45 ± 0.03 ^FGHIc^	6.09 ± 0.11 ^DEFb^	6.11 ± 0.00 ^Fb^	6.13 ± 0.01 ^DEFGb^	6.07 ± 0.04 ^FGb^	6.00 ± 0.01 ^Eb^	5.87 ± 0.02 ^GHa^
T3	6.40 ± 0.02 ^Ab^	6.77 ± 0.04 ^Md^	6.74 ± 0.04 ^Kd^	6.57 ± 0.01 ^Hc^	6.55 ± 0.00 ^Jc^	6.43 ± 0.04 ^FGb^	6.26 ± 0.04 ^HIJa^	6.22 ± 0.01 ^Fa^	6.17 ± 0.04 ^Ia^
T4	6.39 ± 0.01 ^Aabc^	6.56 ± 0.01 ^EFGHIJKc^	6.49 ± 0.01 ^HIJc^	6.45 ± 0.01 ^GHbc^	6.49 ± 0.02 ^IJc^	6.45 ± 0.01 ^FGbc^	6.35 ± 0.03 ^Jab^	6.34 ± 0.08 ^Fa^	6.32 ± 0.01 ^Ja^
T5	6.36 ± 0.00 ^Ab^	6.51 ± 0.01 ^FGHIJKLcd^	6.49 ± 0.01 ^HIJcd^	6.21 ± 0.04 ^Fa^	6.11 ± 0.02 ^Fa^	6.11 ± 0.03 ^CDEFGa^	6.64 ± 0.07 ^Ke^	6.58 ± 0.06 ^Gde^	6.43 ± 0.01 ^Kbc^
T6	6.33 ± 0.04 ^Ae^	6.31 ± 0.01 ^CDe^	6.34 ± 0.03 ^DEFe^	6.26 ± 0.02 ^FGde^	6.12 ± 0.02 ^Fbc^	6.28 ± 0.03 ^FGde^	6.18 ± 0.08 ^HIJcd^	6.01 ± 0.04 ^Eb^	5.81 ± 0.01 ^FGa^
T7	6.37 ± 0.01 ^Ab^	6.62 ± 0.02 ^JKLc^	6.59 ± 0.01 ^Jc^	6.61 ± 0.04 ^Hc^	6.58 ± 0.01 ^Jc^	6.54 ± 0.04 ^FGc^	6.34 ± 0.06 ^IJab^	6.27 ± 0.02 ^Fa^	6.26 ± 0.03 ^Ja^
T8	6.38 ± 0.01 ^Aba^	6.69 ± 0.06 ^KLMb^	6.60 ± 0.08 ^Jb^	6.61 ± 0.04 ^Hb^	6.58 ± 0.02 ^Jb^	6.58 ± 0.09 ^Gb^	6.35 ± 0.00 ^Ja^	6.27 ± 0.06 ^Fa^	6.34 ± 0.02 ^JKa^
T9	6.33 ± 0.04 ^Ad^	6.05 ± 0.07 ^Ac^	6.27 ± 0.04 ^Dd^	6.22 ± 0.14 ^Fcd^	6.36 ± 0.04 ^HId^	6.20 ± 0.01 ^EFGcd^	5.66 ± 0.06 ^Cb^	5.71 ± 0.02 ^Db^	5.22 ± 0.01 ^Ba^
T10	6.36 ± 0.11 ^Ab^	6.41 ± 0.01 ^CDEFGHb^	6.44 ± 0.01 ^EFGHIb^	6.17 ± 0.01 ^Fb^	6.29 ± 0.01 ^GHb^	6.4 ± 0.66 ^FGb^	5.78 ± 0.02 ^CDab^	5.30 ± 0.07 ^Ca^	5.15 ± 0.04 ^Ba^
T11	6.40 ± 0.02 ^Ad^	6.59 ± 0.04 ^JKLf^	6.51 ± 0.05 ^IJef^	6.51 ± 0.04 ^Hef^	6.47 ± 0.03 ^HIde^	6.13 ± 0.01 ^DEFGc^	5.92 ± 0.03 ^DEb^	5.89 ± 0.01 ^Eb^	5.77 ± 0.02 ^EFa^
T12	6.41 ± 0.04 ^Ad^	6.60 ± 0.01 ^KLMe^	6.47 ± 0.00 ^GHId^	6.47 ± 0.01 ^GHd^	6.40 ± 0.03 ^HId^	6.27 ± 0.03 ^FGc^	6.18 ± 0.01 ^GHIc^	6.05 ± 0.07 ^Eb^	5.90 ± 0.04 ^GHa^
T13	6.38 ± 0.01 ^Aef^	6.36 ± 0.06 ^CDEFef^	6.42 ± 0.03 ^EFGHIf^	6.22 ± 0.01 ^Fde^	6.14 ± 0.01 ^Fcd^	6.00 ± 0.15 ^BCDEFbc^	5.88 ± 0.04 ^DEb^	5.68 ± 0.04 ^Da^	5.65 ± 0.01 ^Da^
T14	6.37 ± 0.03 ^Ae^	6.35 ± 0.01 ^CDEe^	6.30 ± 0.00 ^Dde^	6.21 ± 0.02 ^Fcd^	6.19 ± 0.08 ^FGc^	6.01 ± 0.01 ^BCDEFb^	5.94 ± 0.04 ^EFb^	5.60 ± 0.01 ^Da^	5.64 ± 0.01 ^Da^
T15	6.38 ± 0.00 ^Ab^	6.56 ± 0.01 ^IJKLc^	6.43 ± 0.05 ^EFGHIbc^	6.45 ± 0.05 ^GHbc^	6.44 ± 0.01 ^IJbc^	6.37 ± 0.01 ^FGb^	6.11 ± 0.03 ^GHa^	6.02 ± 0.09 ^Ea^	5.95 ± 0.08 ^Ha^
T16	6.40 ± 0.01 ^Aab^	6.60 ± 0.00 ^KLMc^	6.60 ± 0.04 ^Jc^	6.57 ± 0.03 ^Hbc^	6.47 ± 0.13 ^IJabc^	6.42 ± 0.02 ^FGabc^	6.34 ± 0.05 ^IJa^	6.35 ± 0.00 ^Fa^	6.28 ± 0.01 ^Ja^
T17	6.53 ± 0.04 ^Aef^	6.61 ± 0.00 ^KLMf^	6.43 ± 0.01 ^EFGHIe^	6.13 ± 0.07 ^EFd^	5.93 ± 0.07 ^Ec^	5.57 ± 0.03 ^ABa^	5.46 ± 0.02 ^Ba^	5.73 ± 0.02 ^Db^	5.71 ± 0.03 ^DEFb^
T18	6.38 ± 0.00 ^Ad^	6.37 ± 0.01 ^CDEFGd^	5.62 ± 0.01 ^Ac^	5.08 ± 0.01 ^Ab^	5.06 ± 0.06 ^Ab^	5.15 ± 0.04 ^Ab^	5.13 ± 0.02 ^Ab^	4.87 ± 0.06 ^Aa^	4.85 ± 0.01 ^Aa^
T19	6.39 ± 0.01 ^Af^	6.19 ± 0.08 ^Bef^	6.00 ± 0.02 ^Bde^	5.91 ± 0.15 ^CDcd^	5.77 ± 0.04 ^CDbcd^	5.71 ± 0.02 ^BCDEbc^	5.63 ± 0.01 ^Cab^	5.61 ± 0.02 ^Dab^	5.43 ± 0.03 ^Ca^
T20	6.36 ± 0.04 ^Ae^	6.52 ± 0.06 ^HIJKLe^	6.36 ± 0.06 ^DEFGe^	5.83 ± 0.00 ^BCd^	5.80 ± 0.03 ^CDEd^	5.58 ± 0.06 ^ABCc^	5.38 ± 0.05 ^Bb^	5.29 ± 0.08 ^Cab^	5.14 ± 0.06 ^Ba^
T21	6.31 ± 0.04 ^Af^	6.64 ± 0.01 ^LMg^	5.90 ± 0.01 ^Be^	5.74 ± 0.04 ^BCd^	5.61 ± 0.01 ^Bc^	5.67 ± 0.04 ^ABCDEcd^	5.71 ± 0.04 ^Ccd^	5.07 ± 0.03 ^Bb^	4.91 ± 0.01 ^Aa^
T22	6.40 ± 0.01 ^Ade^	6.43 ± 0.00 ^DEFGHIe^	6.29 ± 0.01 ^Dd^	5.66 ± 0.10 ^Bc^	5.70 ± 0.01 ^BCc^	5.63 ± 0.03 ^ABCDc^	5.34 ± 0.06 ^Bb^	5.06 ± 0.01 ^Ba^	5.12 ± 0.01 ^Ba^
T23	6.39 ± 0.01 ^Ag^	6.29 ± 0.05 ^BCfg^	6.16 ± 0.03 ^Cf^	5.94 ± 0.10 ^CDEe^	5.61 ± 0.01 ^Bd^	5.15 ± 0.04 ^Ac^	5.08 ± 0.10 ^Abc^	4.96 ± 0.04 ^Bab^	4.89 ± 0.03 ^Aa^
T24	6.40 ± 0.01 ^Af^	6.51 ± 0.02 ^GHIJKLf^	6.33 ± 0.02 ^Dee^	5.93 ± 0.02 ^CDEd^	5.89 ± 0.02 ^DEd^	5.66 ± 0.08 ^ABCDEc^	5.37 ± 0.04 ^Bb^	5.25 ± 0.05 ^Cab^	5.18 ± 0.03 ^Ba^

Means from each sampling time in the same column with the same uppercase letters are not significantly different (*p* > 0.05). Means from each sampling treatment in the same row with the same lowercase letters are not significantly different (*p* > 0.05). ^1^ Values are the means of 4 replicates (n = 4) ± SD. ^2^ Treatments as described in Table 1. ^3^ Day 0 is 12 h after adding cheese to brine solution.

**Table 3:** pH values ^1^ of brine solution of white-brined cheese inoculated with *L. monocytogenes* or *L. reuteri* and *L. monocytogenes* and stored in 10% or 15% brine solution at 4 °C, 10 °C, and 24 °C for 91 days.

Treatments ^2^	0 ^3^	3	7	14	21	28	49	71	91
T1	6.60 ± 0.04 ^DEFe^	6.68 ± 0.04 ^GHIJe^	6.41 ± 0.05 ^BCDEd^	6.31 ± 0.04 ^CDcd^	6.22 ± 0.01 ^DEc^	6.09 ± 0.04 ^Ab^	5.94 ± 0.03 ^FGa^	5.83 ± 0.04 ^FGHIa^	5.89 ± 0.02 ^CDEFa^
T2	6.71 ± 0.04 ^GHIJf^	6.67 ± 0.01 ^GHIJf^	6.58 ± 0.00 ^FGHIe^	6.22 ± 0.01 ^BCd^	6.20 ± 0.00 ^DEd^	6.10 ± 0.06 ^Ac^	5.92 ± 0.01 ^EFGb^	5.89 ± 0.00 ^GHIab^	5.82 ± 0.02 ^CDFa^
T3	6.71 ± 0.05 ^GHIJd^	6.69 ± 0.08 ^HIJcd^	6.68 ± 0.00 ^Icd^	6.61 ± 0.04 ^Hc^	6.49 ± 0.02 ^FGHb^	6.42 ± 0.03 ^Aab^	6.48 ± 0.01 ^Jb^	6.40 ± 0.01 ^Lab^	6.33 ± 0.02 ^FGa^
T4	6.77 ± 0.00 ^HIJe^	6.68 ± 0.01 ^GHIJe^	6.59 ± 0.03 ^GHIe^	6.40 ± 0.04 ^CDEFd^	6.36 ± 0.06 ^EFGHcd^	6.32 ± 0.05 ^Acd^	6.19 ± 0.03 ^Hbc^	6.12 ± 0.12 ^Kab^	5.96 ± 0.07 ^CDEFGa^
T5	6.57 ± 0.03 ^CDEd^	6.18 ± 0.06 ^Abc^	6.61 ± 0.01 ^GHId^	6.25 ± 0.00 ^BCDc^	6.08 ± 0.08 ^BCD^	6.17 ± 0.01 ^Abc^	5.78 ± 0.06 ^Da^	5.76 ± 0.01 ^EFGa^	5.71 ± 0.04 ^CDEFa^
T6	6.66 ± 0.02 ^EFGf^	6.41 ± 0.00 ^BCDe^	6.45 ± 0.01 ^CDEFe^	6.25 ± 0.06 ^BCDcd^	6.29 ± 0.02 ^EFd^	6.16 ± 0.05 ^Ac^	5.93 ± 0.01 ^EFGb^	5.97 ± 0.04 ^IJb^	5.78 ± 0.04 ^CDEFa^
T7	6.77 ± 0.03 ^HIGc^	6.73 ± 0.02 ^IJc^	6.71 ± 0.07 ^Ic^	6.53 ± 0.03 ^EFGHb^	6.47 ± 0.01 ^FGHb^	6.50 ± 0.01 ^Ab^	6.34 ± 0.00 ^Ia^	6.32 ± 0.01 ^La^	6.36 ± 0.02 ^FGa^
T8	6.81 ± 0.03 ^Jd^	6.75 ± 0.06 ^Jd^	6.71 ± 0.01 ^Id^	6.55 ± 0.06 ^FGHc^	6.52 ± 0.06 ^GHc^	6.47 ± 0.09 ^Abc^	6.33 ± 0.02 ^Iab^	6.29 ± 0.03 ^La^	6.27 ± 0.02 ^EFGa^
T9	6.56 ± 0.04 ^CDd^	6.55 ± 0.01 ^EFGHd^	6.46 ± 0.01 ^CDEFd^	6.31 ± 0.02 ^CDc^	6.36 ± 0.02 ^EFGHc^	6.15 ± 0.04 ^Ab^	5.80 ± 0.01 ^DEa^	5.73 ± 0.04 ^DEFa^	6.11 ± 0.06 ^DEFGb^
T10	6.49 ± 0.03 ^BCa^	6.39 ± 0.01 ^BCba^	6.32 ± 0.04 ^Aba^	6.01 ± 0.15 ^Aa^	5.99 ± 0.08 ^BCa^	5.99 ± 0.01 ^Aa^	5.74 ± 0.01 ^CDa^	5.65 ± 0.00 ^CDEa^	6.08 ± 0.66 ^DEFGa^
T11	6.80 ± 0.01 ^IJe^	6.74 ± 0.06 ^Jde^	6.67 ± 0.02 ^Icde^	6.55 ± 0.03 ^FGHcd^	6.48 ± 0.17 ^FGHc^	6.25 ± 0.04 ^Ab^	5.86 ± 0.02 ^DEFa^	5.80 ± 0.01 ^FGHa^	5.78 ± 0.05 ^CDEFa^
T12	6.77 ± 0.02 ^HIJa^	6.64 ± 0.03 ^FGHIJa^	6.54 ± 0.09 ^EFGHa^	6.43 ± 0.02 ^DEFGa^	6.45 ± 0.02 ^FGHa^	4.85 ± 2.14 ^Aa^	6.16 ± 0.05 ^Ha^	6.09 ± 0.01 ^JKa^	5.96 ± 0.04 ^CDEFGa^
T13	6.73 ± 0.02 ^GHIJf^	6.50 ± 0.01 ^CDEe^	6.65 ± 0.03 ^HIef^	6.36 ± 0.04 ^CDEd^	6.31 ± 0.02 ^EFGD^	5.98 ± 0.06 ^Ac^	5.79 ± 0.06 ^DEb^	5.73 ± 0.07 ^DEFb^	5.53 ± 0.04 ^ABCDa^
T14	6.39 ± 0.04 ^Ade^	6.36 ± 0.03 ^Bde^	6.49 ± 0.01 ^CDEFGe^	6.32 ± 0.02 ^CDd^	6.08 ± 0.05 ^BCDc^	5.91 ± 0.04 ^Ab^	5.64 ± 0.06 ^Ca^	5.58 ± 0.03 ^Ca^	5.59 ± 0.03 ^BCDEa^
T15	6.73 ± 0.03 ^GHIJe^	6.73 ± 0.01 ^IJe^	6.67 ± 0.01 ^Ie^	6.56 ± 0.06 ^FGHd^	6.53 ± 0.03 ^Hd^	6.36 ± 0.02 ^Ac^	6.00 ± 0.01 ^Gb^	5.89 ± 0.01 ^GHIa^	5.90 ± 0.00 ^CDEFa^
T16	6.71 ± 0.03 ^GHIJd^	6.63 ± 0.08 ^EFGHIJd^	6.71 ± 0.01 ^Id^	6.59 ± 0.01 ^GHd^	6.43 ± 0.06 ^FGHc^	6.40 ± 0.00 ^Abc^	6.35 ± 0.00 ^Iabc^	6.30 ± 0.02 ^Lab^	6.24 ± 0.04 ^DEFGa^
T17	6.39 ± 0.02 ^Ae^	6.37 ± 0.04 ^Bde^	6.39 ± 0.02 ^BCDe^	6.26 ± 0.04 ^BCDcd^	6.34 ± 0.04 ^EFGHde^	6.20 ± 0.04 ^Ac^	5.80 ± 0.02 ^DEb^	5.65 ± 0.04 ^CDEa^	6.62 ± 0.03 ^Gf^
T18	6.45 ± 0.04 ^ABe^	6.38 ± 0.03 ^BCe^	6.36 ± 0.03 ^BCe^	6.10 ± 0.08 ^ABd^	6.02 ± 0.02 ^BCDd^	5.94 ± 0.06 ^Acd^	5.83 ± 0.08 ^DEFbc^	5.75 ± 0.00 ^EFGb^	4.89 ± 0.04 ^Aa^
T19	6.74 ± 0.01 ^GHIJg^	6.56 ± 0.05 ^EFGHf^	6.30 ± 0.04 ^ABe^	6.06 ± 0.04 ^Ad^	5.93 ± 0.06 ^ABc^	5.77 ± 0.04 ^Ab^	5.63 ± 0.04 ^Ca^	5.60 ± 0.01 ^CDa^	5.56 ± 0.04 ^BCDEa^
T20	6.76 ± 0.04 ^GHIJf^	6.53 ± 0.02 ^DEFe^	6.24 ± 0.06 ^Ad^	6.00 ± 0.02 ^Ac^	5.77 ± 0.02 ^Ab^	5.77 ± 0.08 ^Ab^	5.65 ± 0.05 ^Cab^	5.58 ± 0.06 ^Ca^	5.53 ± 0.06 ^ABCDa^
T21	6.69 ± 0.01 ^FGHIe^	6.63 ± 0.00 ^EFGHIJe^	6.67 ± 0.04 ^Ie^	6.43 ± 0.02 ^DEFGd^	6.08 ± 0.06 ^BCDc^	5.61 ± 0.04 ^Ab^	5.43 ± 0.04 ^Ba^	5.35 ± 0.01 ^Ba^	5.54 ± 0.06 ^ABCDb^
T22	6.67 ± 0.00 ^FGHf^	6.65 ± 0.01 ^FGHIJef^	6.46 ± 0.02 ^CDEFe^	6.57 ± 0.02 ^FGHd^	6.08 ± 0.05 ^BCDc^	5.57 ± 0.02 ^Ab^	5.03 ± 0.04 ^Aa^	5.07 ± 0.03 ^Aa^	5.02 ± 0.04 ^ABa^
T23	6.70 ± 0.01 ^GHIJa^	6.54 ± 0.01 ^EFGa^	6.38 ± 0.01 ^BCa^	6.36 ± 0.05 ^CDEa^	6.19 ± 0.01 ^CDEa^	6.24 ± 0.03 ^Aa^	5.95 ± 0.07 ^FGa^	5.94 ± 0.07 ^HIa^	6.41 ± 0.57 ^FGa^
T24	6.79 ± 0.02 ^IJf^	6.60 ± 0.04 ^EFGHIe^	6.52 ± 0.02 ^DEFGde^	6.42 ± 0.03 ^DEFGcd^	6.32 ± 0.04 ^EFGHc^	6.13 ± 0.04 ^Ab^	5.35 ± 0.01 ^Ba^	5.25 ± 0.05 ^Ba^	5.27 ± 0.06 ^ABCa^

Means from each sampling time in the same column with the same uppercase letters are not significantly different (*p* > 0.05). Means from each sampling treatment in the same row with the same lowercase letters are not significantly different (*p* > 0.05). ^1^ Values are the means of 4 replicates (n = 4) ± SD. ^2^ Treatments as described in Table 1. ^3^ Day 0 is 12 h after adding cheese to brine solution.

**Table 4:** Water activity values ^1^ of white-brined cheese inoculated with *L. monocytogenes* or *L. reuteri* and *L. monocytogenes* stored in 10% or 15% brine solution at 4 °C for 91 days.

Treatments ^2^	0 ^3^	3	7	14	21	28	49	71	91
T1	0.92 ± 0.01 ^Aab^	0.94 ± 0.01 ^Ab^	0.95 ± 0.01 ^BCb^	0.93 ± 0.01 ^ABab^	0.91 ± 0.01 ^Aa^	0.92 ± 0.00 ^Aab^	0.93 ± 0.00 ^ABab^	0.94 ± 0.01 ^BCDb^	0.94 ± 0.00 ^Ab^
T2	0.92 ± 0.00 ^Aa^	0.95 ± 0.01 ^Aa^	0.93 ± 0.01 ^ABCa^	0.93 ± 0.01 ^ABa^	0.92 ± 0.01 ^Aba^	0.94 ± 0.02 ^Aa^	0.95 ± 0.01 ^Ba^	0.95 ± 0.00 ^Da^	0.95 ± 0.01 ^Aa^
T3	0.96 ± 0.01 ^Aa^	0.94 ± 0.01 ^Aa^	0.94 ± 0.01 ^ABCa^	0.94 ± 0.00 ^ABa^	0.95 ± 0.01 ^Ba^	0.94 ± 0.01 ^Aa^	0.95 ± 0.01 ^Ba^	0.94 ± 0.00 ^CDa^	0.94 ± 0.00 ^Aa^
T4	0.94 ± 0.00 ^Aa^	0.94 ± 0.00 ^Aa^	0.94 ± 0.00 ^BCa^	0.95 ± 0.01 ^ABa^	0.95 ± 0.01 ^Ba^	0.95 ± 0.01 ^Aa^	0.95 ± 0.01 ^Ba^	0.94 ± 0.01 ^BCDa^	0.94 ± 0.00 ^Aa^
T5	0.94 ± 0.00 ^Aa^	0.93 ± 0.01 ^Aa^	0.92 ± 0.02 ^ABCa^	0.92 ± 0.01 ^Aa^	0.93 ± 0.02 ^ABa^	0.91 ± 0.01 ^Aa^	0.93 ± 0.01 ^ABa^	0.93 ± 0.00 ^ABCDa^	0.92 ± 0.01 ^Aa^
T6	0.93 ± 0.01 ^Aa^	0.92 ± 0.01 ^Aa^	0.93 ± 0.01 ^ABCa^	0.92 ± 0.01 ^ABa^	0.94 ± 0.01 ^ABa^	0.94 ± 0.01 ^Aa^	0.92 ± 0.01 ^ABa^	0.91 ± 0.01 ^ABCa^	0.90 ± 0.04 ^Aa^
T7	0.94 ± 0.01 ^Aa^	0.92 ± 0.01 ^Aa^	0.93 ± 0.00 ^ABCa^	0.93 ± 0.01 ^ABa^	0.93 ± 0.00 ^ABa^	0.95 ± 0.01 ^Aa^	0.93 ± 0.01 ^ABa^	0.94 ± 0.01 ^BCDa^	0.93 ± 0.00 ^Aa^
T8	0.94 ± 0.01 ^Aa^	0.93 ± 0.01 ^Aa^	0.93 ± 0.01 ^ABCa^	0.94 ± 0.01 ^ABa^	0.93 ± 0.01 ^ABa^	0.93 ± 0.01 ^Aa^	0.92 ± 0.01 ^ABa^	0.93 ± 0.01 ^ABCDa^	0.93 ± 0.01 ^Aa^
T9	0.96 ± 0.01 ^Ab^	0.94 ± 0.01 ^Aab^	0.92 ± 0.01 ^ABCba^	0.93 ± 0.01 ^ABab^	0.94 ± 0.01 ^ABab^	0.95 ± 0.00 ^Ab^	0.95 ± 0.01 ^ABab^	0.94 ± 0.00 ^CDab^	0.95 ± 0.01 ^Aab^
T10	0.93 ± 0.01 ^Aa^	0.93 ± 0.01 ^Aa^	0.94 ± 0.01 ^ABCa^	0.93 ± 0.01 ^ABa^	0.93 ± 0.01 ^ABa^	0.93 ± 0.01 ^Aa^	0.91 ± 0.01 ^Ba^	0.92 ± 0.01 ^ABCDa^	0.93 ± 0.01 ^Aa^
T11	0.94 ± 0.00 ^Aa^	0.95 ± 0.01 ^Aa^	0.94 ± 0.00 ^BCa^	0.94 ± 0.01 ^ABa^	0.94 ± 0.01 ^ABa^	0.94 ± 0.01 ^Aa^	0.94 ± 0.01 ^ABa^	0.94 ± 0.00 ^CDa^	0.93 ± 0.00 ^Aa^
T12	0.94 ± 0.00 ^Aa^	0.95 ± 0.01 ^Aa^	0.94 ± 0.01 ^ABCa^	0.94 ± 0.01 ^ABa^	0.94 ± 0.01 ^ABa^	0.94 ± 0.00 ^Aa^	0.95 ± 0.01 ^Ba^	0.94 ± 0.01 ^BCDa^	0.93 ± 0.02 ^Aa^
T13	0.93 ± 0.03 ^Aa^	0.92 ± 0.01 ^Aa^	0.94 ± 0.01 ^ABCa^	0.93 ± 0.01 ^ABa^	0.92 ± 0.01 ^ABa^	0.93 ± 0.01 ^Aa^	0.93 ± 0.01 ^ABa^	0.91 ± 0.01 ^ABa^	0.93 ± 0.01 ^Aa^
T14	0.93 ± 0.01 ^Aa^	0.94 ± 0.01 ^Aa^	0.90 ± 0.00 ^Aa^	0.93 ± 0.01 ^ABa^	0.93 ± 0.01 ^ABa^	0.92 ± 0.02 ^Aa^	0.93 ± 0.01 ^ABa^	0.90 ± 0.01 ^Aa^	0.95 ± 0.02 ^Aa^
T15	0.93 ± 0.01 ^Aa^	0.94 ± 0.01 ^Aa^	0.93 ± 0.01 ^ABCa^	0.93 ± 0.01 ^ABa^	0.92 ± 0.01 ^ABa^	0.93 ± 0.01 ^Aa^	0.92 ± 0.01 ^ABa^	0.94 ± 0.0 ^BCDa^	0.93 ± 0.01 ^Aa^
T16	0.94 ± 0.01 ^Aa^	0.93 ± 0.01 ^Aa^	0.93 ± 0.01 ^ABCa^	0.94 ± 0.01 ^ABa^	0.93 ± 0.01 ^ABa^	0.94 ± 0.01 ^Aa^	0.91 ± 0.01 ^ABa^	0.91 ± 0.00 ^ABCa^	0.93 ± 0.01 ^Aa^
T17	0.95 ± 0.01 ^Aa^	0.94 ± 0.00 ^Aa^	0.94 ± 0.01 ^BCa^	0.94 ± 0.01 ^ABa^	0.95 ± 0.02 ^Ba^	0.92 ± 0.00 ^Aa^	0.92 ± 0.01 ^ABa^	0.93 ± 0.01 ^ABCDa^	0.93 ± 0.00 ^Aa^
T18	0.95 ± 0.01 ^Aa^	0.93 ± 0.01 ^Aa^	0.94 ± 0.00 ^BCa^	0.93 ± 0.00 ^ABa^	0.94 ± 0.01 ^ABa^	0.94 ± 0.00 ^Aa^	0.94 ± 0.01 ^ABa^	0.94 ± 0.01 ^BCDa^	0.94 ± 0.00 ^Aa^
T19	0.94 ± 0.01 ^Aa^	0.94 ± 0.00 ^Aa^	0.94 ± 0.01 ^ABCa^	0.95 ± 0.01 ^ABa^	0.94 ± 0.01 ^ABa^	0.94 ± 0.01 ^Aa^	0.93 ± 0.00 ^ABa^	0.93 ± 0.00 ^ABCDa^	0.94 ± 0.01 ^Aa^
T20	0.95 ± 0.01 ^Aa^	0.95 ± 0.01 ^Aa^	0.94 ± 0.00 ^BCa^	0.94 ± 0.00 ^ABa^	0.95 ± 0.01 ^Ba^	0.93 ± 0.01 ^Aa^	0.94 ± 0.01 ^ABa^	0.94 ± 0.01 ^CDa^	0.94 ± 0.00 ^Aa^
T21	0.94 ± 0.01 ^Aab^	0.93 ± 0.01 ^Aab^	0.95 ± 0.01 ^Cb^	0.94 ± 0.01 ^ABab^	0.93 ± 0.00 ^ABab^	0.95 ± 0.01 ^Aab^	0.92 ± 0.01 ^ABa^	0.92 ± 0.01 ^ABCDab^	0.93 ± 0.00 ^Aab^
T22	0.94 ± 0.02 ^Aa^	0.94 ± 0.01 ^Aa^	0.95 ± 0.01 ^BCa^	0.96 ± 0.02 ^Ba^	0.94 ± 0.00 ^ABa^	0.93 ± 0.01 ^Aa^	0.90 ± 0.04 ^Aa^	0.94 ± 0.01 ^BCDa^	0.93 ± 0.00 ^Aa^
T23	0.93 ± 0.01 ^Aab^	0.93 ± 0.01 ^Aab^	0.91 ± 0.01 ^ABa^	0.93 ± 0.01 ^ABab^	0.95 ± 0.01 ^Bb^	0.94 ± 0.01 ^Aab^	0.91 ± 0.01 ^ABa^	0.92 ± 0.00 ^ABCDab^	0.94 ± 0.01 ^Aab^
T24	0.94 ± 0.01 ^Aa^	0.92 ± 0.01 ^Aa^	0.93 ± 0.00 ^ABCa^	0.92 ± 0.01 ^ABa^	0.92 ± 0.01 ^ABa^	0.94 ± 0.01 ^Aa^	0.92 ± 0.01 ^ABa^	0.93 ± 0.00 ^ABCDa^	0.94 ± 0.01 ^Aa^

Means from each sampling time in the same column with the same uppercase letters are not significantly different (*p* > 0.05). Means from each sampling treatment in the same row with the same lowercase letters are not significantly different (*p* > 0.05). ^1^ Values are the means of 4 replicates (n = 4) ± SD. ^2^ Treatments as described in Table 1. ^3^ Day 0 is 12 h after adding cheese to brine solution.

**Table 5:** Salt content ^1^ of white-brined cheese in the presence of *L. monocytogenes* or *L. reuteri* and *L. monocytogenes* and stored in 10% or 15% brine solution at 4 10, or 24 °C for 91 days.

Storage Time (Days)									
Treatments ^2^	0 ^3^	3	7	14	21	28	49	71	91
T1	4.75 ± 0.05 ^ABCDa^	5.38 ± 0.05 ^ABb^	5.85 ± 0.09 ^ABCc^	6.10 ± 0.01 ^Bcd^	6.06 ± 0.11 ^Bcd^	5.90 ± 0.19 ^Bc^	5.78 ± 0.13 ^Ac^	6.26 ± 0.04 ^Bd^	6.25 ± 0.06 ^ABd^
T2	5.79 ± 0.12 ^HIa^	6.82 ± 0.13 ^GHIb^	7.00 ± 0.14 ^GHIb^	6.80 ± 0.03 ^CDEFb^	6.72 ± 0.06 ^EFGb^	6.75 ± 0.06 ^EFGb^	6.74 ± 0.06 ^FGHIb^	6.90 ± 0.01 ^GHIb^	6.75 ± 0.03 ^ABCb^
T3	6.10 ± 0.01 ^Ia^	5.91 ± 0.02 ^BCDEa^	6.74 ± 0.01 ^EFGbc^	7.06 ± 0.24 ^DEFc^	6.84 ± 0.13 ^EFbc^	6.72 ± 0.04 ^EFGbc^	6.82 ± 0.06 ^GHIbc^	6.76 ± 0.03 ^EFGHbc^	6.68 ± 0.04 ^ABCb^
T4	6.10 ± 0.16 ^Ia^	6.38 ± 0.10 ^EFGHab^	6.82 ± 0.04 ^FGc^	6.80 ± 0.11 ^CDEFc^	6.72 ± 0.09 ^EFGc^	6.86 ± 0.04 ^GHc^	6.68 ± 0.05 ^FGHIbc^	6.79 ± 0.13 ^EFGHc^	6.78 ± 0.05 ^BCc^
T5	4.49 ± 0.04 ^ABa^	6.08 ± 0.09 ^CDEFb^	6.85 ± 0.23 ^FGd^	7.21 ± 0.05 ^Fe^	6.80 ± 0.05 ^FGcd^	6.47 ± 0.06 ^DEc^	6.69 ± 0.04 ^FGHIcd^	6.79 ± 0.08 ^EFGHcd^	6.77 ± 0.03 ^BCcd^
T6	5.29 ± 0.08 ^CDEFGHa^	6.90 ± 0.16 ^HIde^	7.20 ± 0.01 ^HIe^	6.82 ± 0.08 ^CDEd^	6.88 ± 0.18 ^FGde^	6.81 ± 0.06 ^GHd^	6.11 ± 0.01 ^Bb^	6.35 ± 0.11 ^BCbc^	6.64 ± 0.04 ^ABCcd^
T7	5.36 ± 0.07 ^DEFGHa^	6.39 ± 0.11 ^EFGHb^	6.73 ± 0.08 ^EFGc^	6.71 ± 0.10 ^CDEFc^	6.71 ± 0.03 ^EFc^	6.80 ± 0.02 ^EFGc^	6.71 ± 0.04 ^FGHIc^	6.61 ± 0.04 ^CDEFbc^	6.83 ± 0.12 ^BCc^
T8	4.27 ± 0.08 ^Aa^	5.44 ± 0.04 ^ABCb^	6.17 ± 0.08 ^CDc^	6.49 ± 0.08 ^BCd^	6.68 ± 0.05 ^EFde^	6.70 ± 0.12 ^EFGde^	6.81 ± 0.03 ^GHIe^	6.79 ± 0.04 ^EFGHe^	6.62 ± 0.04 ^ABde^
T9	5.68 ± 0.09 ^HIa^	5.71 ± 0.02 ^ABCDa^	5.52 ± 0.04 ^Aa^	5.66 ± 0.04 ^Aa^	6.15 ± 0.06 ^BCb^	6.04 ± 0.08 ^BCb^	6.25 ± 0.08 ^BCDb^	6.25 ± 0.04 ^Bb^	6.30 ± 0.16 ^ABb^
T10	5.71 ± 0.04 ^HIa^	6.19 ± 0.76 ^DEFGab^	6.73 ± 0.12 ^EFGb^	7.06 ± 0.04 ^DEFb^	6.82 ± 0.04 ^FGb^	6.75 ± 0.03 ^EFGb^	6.81 ± 0.04 ^GHIb^	6.82 ± 0.04 ^FGHb^	6.92 ± 0.25 ^BCb^
T11	5.59 ± 0.04 ^GHIa^	6.59 ± 0.14 ^FGHIb^	7.03 ± 0.16 ^GHIc^	6.59 ± 0.04 ^BCDEb^	6.76 ± 0.04 ^FGbc^	6.63 ± 0.03 ^EFGb^	6.64 ± 0.13 ^EFGHb^	6.8 ± 0.01 ^EFGHbc^	6.75 ± 0.05 ^ABCbc^
T12	5.55 ± 0.01 ^FGHIa^	5.69 ± 0.05 ^ABCDa^	6.74 ± 0.06 ^EFGc^	6.78 ± 0.08 ^CDEFc^	6.20 ± 0.01 ^BCDb^	6.92 ± 0.04 ^GHcd^	6.88 ± 0.1 ^OHIc^	7.11 ± 0.02 ^Id^	6.83 ± 0.09 ^BCc^
T13	5.19 ± 0.04 ^BCDEFGHa^	5.45 ± 0.05 ^ABCa^	6.1 ± 0.02 ^BCDb^	6.56 ± 0.13 ^CDEc^	7.01 ± 0.05 ^Gd^	6.72 ± 0.09 ^EFGcd^	6.88 ± 0.05 ^HId^	6.94 ± 0.16 ^GHIcd^	6.80 ± 0.05 ^BCcd^
T14	4.97 ± 0.1 ^BCDEFGa^	5.52 ± 0.08 ^ABCb^	5.98 ± 0.09 ^BCc^	6.68 ± 0.04 ^CDEFde^	6.9 ± 0.04 ^FGe^	6.78 ± 0.09 ^EFGde^	6.8 ± 0.08 ^GHIde^	6.78 ± 0.05 ^EFGHde^	6.61 ± 0.09 ^ABCd^
T15	5.16 ± 0.06 ^BCDEFGHa^	5.37 ± 0.08 ^ABa^	5.78 ± 0.13 ^ABb^	6.46 ± 0.06 ^BCc^	6.68 ± 0.07 ^EFcd^	6.85 ± 0.09 ^GHd^	6.61 ± 0.04 ^EFGHcd^	6.65 ± 0.03 ^DEFGcd^	6.61 ± 0.06 ^ABCcd^
T16	4.89 ± 0.03 ^ABCDEFa^	5.58 ± 0.05 ^ABCDb^	6.16 ± 0.08 ^CDc^	6.46 ± 0.11 ^BCd^	6.67 ± 0.08 ^EFde^	6.65 ± 0.12 ^EFGde^	6.53 ± 0.11 ^EFGde^	6.79 ± 0.03 ^EFGHe^	6.56 ± 0.11 ^ABCde^
T17	5.49 ± 0.08 ^EFGHIa^	6.91 ± 0.03 ^HId^	5.54 ± 0.13 ^Aa^	5.51 ± 0.06 ^Aa^	5.65 ± 0.05 ^Aab^	5.90 ± 0.10 ^Bbc^	5.99 ± 0.03 ^ABc^	5.95 ± 0.11 ^Ac^	6.04 ± 0.11 ^Ac^
T18	4.65 ± 0.79 ^ABCa^	6.80 ± 0.10 ^GHIb^	6.65 ± 0.05 ^EFGb^	6.53 ± 0.07 ^BCDb^	6.45 ± 0.06 ^DEb^	6.28 ± 0.10 ^CDb^	6.38 ± 0.05 ^CDEb^	6.61 ± 0.05 ^CDEFb^	6.56 ± 0.04 ^ABCb^
T19	4.79 ± 0.11 ^ABCDEa^	5.06 ± 0.04 ^Ab^	6.82 ± 0.13 ^FGf^	6.80 ± 0.05 ^CDEFef^	6.25 ± 0.08 ^BCDc^	6.52 ± 0.04 ^DEFcde^	6.46 ± 0.10 ^DEFcd^	6.52 ± 0.06 ^CDEcde^	6.68 ± 0.08 ^ABCdef^
T20	4.55 ± 0.08 ^ABa^	5.30 ± 0.01 ^ABb^	6.70 ± 0.04 ^EFGcd^	6.54 ± 0.16 ^BCDc^	6.68 ± 0.06 ^EFcd^	6.77 ± 0.06 ^EFGcd^	6.96 ± 0.07 ^Id^	6.49 ± 0.11 ^BCDc^	6.62 ± 0.08 ^ABCc^
T21	4.99 ± 0.15 ^BCDEFGa^	6.97 ± 0.06 ^HIJd^	7.31 ± 0.11 ^Ie^	7.12 ± 0.06 ^EFde^	6.87 ± 0.06 ^FGd^	5.99 ± 0.06 ^Bb^	6.16 ± 0.09 ^BCb^	6.83 ± 0.06 ^FGHcd^	6.59 ± 0.04 ^ABCc^
T22	5.19 ± 0.11 ^BCDEFGHa^	7.13 ± 0.15 ^IJc^	6.57 ± 0.11 ^EFbc^	7.11 ± 0.54 ^EFc^	6.17 ± 0.06 ^BCb^	5.02 ± 0.11 ^Aa^	6.77 ± 0.10 ^GHIbc^	6.79 ± 0.06 ^EFGHbc^	6.88 ± 0.08 ^BCbc^
T23	4.79 ± 0.11 ^ABCDEa^	7.23 ± 0.10 ^IJd^	6.96 ± 0.10 ^FGHIc^	6.45 ± 0.08 ^BCb^	6.39 ± 0.11 ^CDb^	6.78 ± 0.04 ^EFGc^	6.18 ± 0.05 ^BCb^	6.43 ± 0.05 ^BCDb^	6.47 ± 0.05 ^ABb^
T24	4.89 ± 0.09 ^ABCDEFa^	7.55 ± 0.13 ^Jc^	6.40 ± 0.11 ^DEb^	6.59 ± 0.03 ^BCDbc^	6.69 ± 0.04 ^EFbc^	7.12 ± 0.13 ^Hbc^	6.81 ± 0.06 ^GHIbc^	7.03 ± 0.13 ^HIbc^	7.28 ± 0.77 ^Cbc^

Means from each sampling time in the same column with the same uppercase letters are not significantly different (*p* > 0.05). Means from each sampling treatment in the same row with the same lowercase letters are not significantly different (*p* > 0.05). ^1^ Values are the means of 4 replicates (n = 4) ± SD. ^2^ Treatments as described in Table 1. ^3^ Day 0 is 12 h after adding cheese to brine solution.

**Table 6:** Survival of *L. reuteri* (log CFU/g) ^1^ in WBC stored in 10% or 15% NaCl brine in the presence of *L. monocytogenes* at 4, 10, and 24 °C for 91 days.

Treatments ^2^	D0 ^3^	D3	D7	D14	D21	D28	D49	D71	D91
T2	6.11 ± 0.03 ^Aa^	6.59 ± 0.40 ^CDab^	6.72 ± 0.44 ^CDEbcd^	6.21 ± 0.03 ^BCab^	6.72 ± 0.29 ^Hbcd^	7.29 ± 0.65 ^FGe^	7.13 ± 0.11 ^Hcd^	7.25 ± 0.12 ^Ede^	7.30 ± 0.09 ^De^
T3	6.76 ± 0.10 ^Cbcd^	6.51 ± 0.42 ^CDbc^	6.99 ± 0.04 ^DEFd^	6.05 ± 0.06 ^Ba^	6.04 ± 0.05 ^EFa^	6.80 ± 0.08 ^EFcd^	6.43 ± 0.01 ^Deb^	6.70 ± 0.13 ^Dbcd^	6.70 ± 0.07 ^Cbcd^
T4	6.49 ± 0.09 ^Bbc^	6.77 ± 0.31 ^Dd^	7.07 ± 0.04 ^EFe^	6.10 ± 0.02 ^Ba^	6.05 ± 0.02 ^EFa^	6.78 ± 0.14 ^EFd^	6.43 ± 0.02 ^Deb^	6.71 ± 0.05 ^Dcd^	6.80 ± 0.06 ^Cd^
T6	6.07 ± 0.04 ^Aab^	5.82 ± 0.11 ^Aba^	6.58 ± 0.53 ^BCDEbcd^	6.45 ± 0.09 ^CDbcd^	5.76 ± 0.12 ^DEFa^	6.73 ± 0.60 ^EFd^	6.68 ± 0.29 ^EFGcd^	6.66 ± 0.19 ^Dcd^	6.15 ± 0.08 ^Babc^
T7	6.69 ± 0.05 ^Ce^	6.14 ± 0.06 ^ABCd^	5.96 ± 0.02 ^Acd^	6.11 ± 0.22 ^Bd^	5.34 ± 0.06 ^BCDa^	6.16 ± 0.02 ^CDEd^	5.83 ± 0.01 ^BCc^	5.49 ± 0.08 ^Aab^	5.63 ± 0.12 ^Ab^
T8	6.69 ± 0.14 ^Cc^	6.41 ± 0.22 ^CDbc^	6.27 ± 0.15 ^ABCb^	5.60 ± 0.36 ^Aa^	5.35 ± 0.02 ^BCDa^	6.07 ± 0.02 ^CDEb^	5.68 ± 0.03 ^Aba^	5.63 ± 0.08 ^Aa^	5.51 ± 0.19 ^Aa^
T10	6.11 ± 0.03 ^Aa^	6.03 ± 0.33 ^Da^	6.21 ± 0.15 ^ABCab^	6.15 ± 0.02 ^Ba^	8.23 ± 0.29 ^Jc^	6.35 ± 0.45 ^CDEab^	6.36 ± 0.26 ^DEab^	6.25 ± 0.12 ^Bab^	6.69 ± 0.04 ^Cb^
T11	6.76 ± 0.03 ^Ad^	6.38 ± 0.46 ^ABc^	6.17 ± 0.23 ^ABCb^	6.27 ± 0.15 ^BCbc^	6.20 ± 0.47 ^FJbc^	5.91 ± 0.19 ^BCDa^	6.15 ± 0.42 ^CDb^	6.30 ± 0.19 ^Babc^	6.12 ± 0.19 ^Bb^
T12	6.49 ± 0.10 ^Cc^	6.41 ± 0.11 ^BCc^	6.41 ± 0.16 ^ABCDc^	6.18 ± 0.02 ^BCba^	6.22 ± 0.13 ^FGab^	6.40 ± 0.07 ^CDEc^	6.43 ± 0.04 ^Dec^	6.34 ± 0.02 ^BCbc^	6.43 ± 0.02 ^BCc^
T14	6.07 ± 0.04 ^Abc^	5.66 ± 0.36 ^Aab^	7.03 ± 0.57 ^EFd^	6.15 ± 0.11 ^Bc^	5.31 ± 0.20 ^BCDa^	5.71 ± 0.19 ^ABCab^	5.37 ± 0.15 ^ABc^	5.30 ± 0.04 ^Bc^	6.19 ± 0.11 ^Bc^
T15	6.69 ± 0.05 ^Cd^	6.34 ± 0.13 ^BCDc^	6.26 ± 0.07 ^ABCc^	5.68 ± 0.05 ^Ab^	5.23 ± 0.19 ^BCa^	5.37 ± 0.14 ^ABa^	5.34 ± 0.04 ^Aa^	5.37 ± 0.03 ^Aa^	5.28 ± 0.08 ^Aa^
T16	6.69 ± 0.14 ^Cd^	6.30 ± 0.03 ^BCDc^	6.42 ± 0.06 ^ABCDc^	5.60 ± 0.15 ^Ab^	5.11 ± 0.17 ^Ba^	5.37 ± 0.14 ^ABb^	5.41 ± 0.06 ^Ab^	5.38 ± 0.04 ^Ab^	5.35 ± 0.15 ^Ab^
T18	6.11 ± 0.03 ^Aa^	7.29 ± 0.16 ^Ede^	7.80 ± 0.17 ^Gf^	7.11 ± 0.03 ^Fcde^	7.38 ± 0.34 ^Ie^	7.71 ± 0.22 ^Gf^	6.88 ± 0.29 ^GHbc^	7.03 ± 0.10 ^Ebcd^	6.71 ± 0.21 ^Cb^
T19	6.76 ± 0.10 ^Cab^	7.42 ± 0.23 ^Eb^	7.35 ± 0.06 ^FGb^	6.58 ± 0.10 ^Da^	6.78 ± 0.19 ^Iab^	6.55 ± 0.52 ^DEa^	6.84 ± 0.18 ^FGHab^	6.62 ± 0.25 ^CDa^	6.64 ± 0.58 ^Ca^
T20	6.49 ± 0.09 ^Ba^	7.35 ± 0.05 ^Ec^	7.43 ± 0.06 ^FGc^	6.83 ± 0.04 ^Eb^	6.63 ± 0.22 ^GHab^	6.31 ± 0.16 ^CDEa^	6.47 ± 0.00 ^DEFa^	6.45 ± 0.30 ^BCDa^	6.44 ± 0.14 ^BCa^
T22	6.07 ± 0.04 ^Ab^	6.44 ± 0.15 ^CDc^	6.67 ± 0.18 ^BCDEc^	5.72 ± 0.05 ^Aab^	5.65 ± 0.36 ^CDEa^	8.39 ± 0.48 ^Hd^	5.50 ± 0.17 ^Aba^	5.40 ± 0.04 ^Aa^	5.35 ± 0.16 ^Aa^
T23	6.69 ± 0.05 ^Cf^	6.33 ± 0.17 ^BCDe^	6.07 ± 0.04 ^ABde^	5.79 ± 0.21 ^Acd^	4.84 ± 0.05 ^ABa^	5.20 ± 0.17 ^Ad^	5.57 ± 0.19 ^ABc^	5.49 ± 0.17 ^Adc^	5.58 ± 0.23 ^Ac^
T24	6.69 ± 0.14 ^Cd^	6.38 ± 0.06 ^BCDd^	5.93 ± 0.08 ^Ac^	5.57 ± 0.08 ^Ab^	4.58 ± 0.19 ^Aa^	5.29 ± 0.17 ^ABb^	5.48 ± 0.17 ^ABb^	5.40 ± 0.29 ^Ab^	5.47 ± 0.15 ^Ab^

Means from each sampling time in the same column with the same uppercase letters are not significantly different (*p* > 0.05). Means from each sampling treatment in the same row with the same lowercase letters are not significantly different (*p* > 0.05). ^1^ Values are the means of 4 replicates (n = 4) ± SD. ^2^ Treatments as described in Table 1. ^3^ Day 0 is 12 h after adding cheese to brine solution.

**Table 7:** Survival of *L. reuteri* (log CFU/g) ^1^ in cheese brine stored in 10% or 15% NaCl brine in the presence of *L. monocytogenes* at 4, 10, and 24 °C for 91 days.

Treatments ^2^	D0 ^3^	D3	D7	D14	D21	D28	D49	D71	D91
T2	6.45 ± 0.02 ^ABa^	6.56 ± 0.50 ^Ca^	7.18 ± 0.12 ^EFb^	6.58 ± 0.07 ^Ja^	7.26 ± 0.28 ^Cb^	7.06 ± 0.10 ^FGb^	7.28 ± 0.14 ^DEb^	7.37 ± 0.05 ^Eb^	7.27 ± 0.02 ^DEb^
T3	6.15 ± 0.17 ^Aa^	7.41 ± 0.06 ^De^	7.16 ± 0.21 ^Ed^	6.33 ± 0.01 ^FGHab^	6.16 ± 0.16 ^Bab^	6.85 ± 0.05 ^EFc^	6.40 ± 0.07 ^BCb^	6.20 ± 0.03 ^CDab^	6.18 ± 0.02 ^Bab^
T4	6.20 ± 0.20 ^Aa^	7.31 ± 0.04 ^Dc^	7.12 ± 0.05 ^Ec^	6.17 ± 0.03 ^EFa^	6.26 ± 0.02 ^Ba^	6.68 ± 0.10 ^DEFb^	6.35 ± 0.21 ^Ba^	6.35 ± 0.07 ^Da^	6.21 ± 0.02 ^Ba^
T6	6.35 ± 0.01 ^ABCa^	6.53 ± 0.37 ^BCa^	6.44 ± 0.44 ^BCDa^	6.21 ± 0.02 ^EFGa^	6.41 ± 0.37 ^Ba^	6.26 ± 0.07 ^Da^	6.47 ± 0.35 ^BCa^	6.35 ± 0.04 ^Da^	6.37 ± 0.13 ^Ba^
T7	6.29 ± 0.14 ^ABd^	6.49 ± 0.34 ^BCd^	5.92 ± 0.07 ^Ac^	6.45 ± 0.09 ^HIJd^	5.15 ± 0.19 ^Aa^	5.57 ± 0.09 ^BCbc^	5.74 ± 0.09 ^Abc^	5.58 ± 0.23 ^ABbc^	5.51 ± 0.05 ^Ab^
T8	6.58 ± 0.16 ^Bd^	6.40 ± 0.23 ^ABCcd^	6.17 ± 0.17 ^ABCc^	6.27 ± 0.14 ^EFGHc^	5.27 ± 0.14 ^Aa^	5.61 ± 0.04 ^BCb^	5.53 ± 0.05 ^Aab^	5.58 ± 0.08 ^ABab^	5.46 ± 0.14 ^Aab^
T10	6.45 ± 0.02 ^Aba^	6.37 ± 0.23 ^ABCa^	8.25 ± 0.11 ^Gc^	6.14 ± 0.04 ^Ea^	6.18 ± 0.10 ^Ba^	6.43 ± 0.39 ^DEa^	6.88 ± 0.41 ^CDb^	6.45 ± 0.17 ^Da^	7.13 ± 0.03 ^Db^
T11	6.15 ± 0.17 ^Aa^	6.40 ± 0.22 ^ABCab^	6.53 ± 0.11 ^CDbc^	6.10 ± 0.03 ^Ea^	6.13 ± 0.04 ^Ba^	6.26 ± 0.05 ^Dab^	6.43 ± 0.04 ^BCab^	6.35 ± 0.28 ^Dab^	6.35 ± 0.12 ^Cab^
T12	6.20 ± 0.20 ^Aab^	6.20 ± 0.20 ^ABCab^	6.05 ± 0.21 ^ABba^	6.37 ± 0.03 ^GHIb^	6.34 ± 0.18 ^Bab^	6.43 ± 0.04 ^DEb^	6.46 ± 0.02 ^BCb^	6.46 ± 0.03 ^Db^	6.30 ± 0.12 ^Cab^
T14	6.35 ± 0.01 ^ABd^	5.91 ± 0.34 ^Abcd^	6.26 ± 0.13 ^ABCcd^	5.12 ± 0.03 ^Aa^	6.27 ± 0.07 ^Bcd^	5.59 ± 0.42 ^BCab^	5.85 ± 0.47 ^Abcd^	5.73 ± 0.59 ^BCbc^	6.22 ± 0.04 ^Bcd^
T15	6.29 ± 0.14 ^ABc^	6.13 ± 0.09 ^ABCc^	6.07 ± 0.06 ^ABc^	5.65 ± 0.06 ^CDb^	5.28 ± 0.28 ^Aab^	5.23 ± 0.22 ^ABa^	5.39 ± 0.06 ^Aab^	5.23 ± 0.28 ^ABba^	5.41 ± 0.14 ^Aab^
T16	6.58 ± 0.16 ^Bc^	6.33 ± 0.10 ^ABCc^	5.97 ± 0.03 ^Ab^	5.51 ± 0.18 ^BCa^	5.44 ± 0.10 ^Aa^	5.10 ± 0.14 ^Aa^	5.42 ± 0.16 ^Aa^	5.13 ± 0.41 ^Aa^	5.36 ± 0.13 ^Aa^
T18	6.45 ± 0.02 ^ABa^	8.27 ± 0.13 ^Ee^	7.55 ± 0.19 ^Fbcd^	6.43 ± 0.10 ^HIJa^	7.84 ± 0.37 ^Dd^	7.33 ± 0.11 ^Gb^	7.65 ± 0.11 ^Ecd^	7.57 ± 0.23 ^Ebcd^	7.48 ± 0.10 ^Ebc^
T19	6.15 ± 0.17 ^Aa^	7.37 ± 0.18 ^Dd^	7.34 ± 0.12 ^EFd^	6.93 ± 0.08 ^Kc^	6.20 ± 0.15 ^Ba^	6.51 ± 0.12 ^DEb^	6.87 ± 0.12 ^CDc^	6.75 ± 0.15 ^Dbc^	6.81 ± 0.06 ^Cc^
T20	6.20 ± 0.20 ^Aba^	7.42 ± 0.06 ^Dd^	7.43 ± 0.03 ^EFd^	6.52 ± 0.06 ^IJbc^	6.34 ± 0.02 ^Bab^	6.54 ± 0.16 ^DEbc^	6.80 ± 0.07 ^BCc^	6.62 ± 0.25 ^Dbc^	6.75 ± 0.11 ^Cc^
T22	6.35 ± 0.01 ^ABb^	6.55 ± 0.17 ^Cb^	6.69 ± 0.25 ^Db^	5.48 ± 0.18 ^BCa^	6.51 ± 0.18 ^Bb^	5.76 ± 0.28 ^Ca^	5.73 ± 0.22 ^Aa^	5.65 ± 0.32 ^ABa^	5.61 ± 0.27 ^Aa^
T23	6.29 ± 0.14 ^ABe^	5.97 ± 0.04 ^ABd^	6.26 ± 0.10 ^ABCe^	5.44 ± 0.01 ^Bbc^	5.19 ± 0.06 ^Aa^	5.36 ± 0.07 ^ABCab^	5.58 ± 0.08 ^Ac^	5.51 ± 0.18 ^ABbc^	5.48 ± 0.04 ^Abc^
T24	6.58 ± 0.16 ^Bd^	6.12 ± 0.09 ^ABCc^	6.07 ± 0.07 ^ABc^	5.81 ± 0.07 ^Dbc^	5.00 ± 0.36 ^Aa^	5.53 ± 0.15 ^BCb^	5.57 ± 0.08 ^Ab^	5.57 ± 0.20 ^ABb^	5.61 ± 0.13 ^Ab^

Means from each sampling time in the same column with the same uppercase letters are not significantly different (*p* > 0.05). Means from each sampling treatment in the same row with the same lowercase letters are not significantly different (*p* > 0.05). ^1^ Values are the means of 4 replicates (n = 4) ± SD. ^2^ Treatments as described in Table 1. ^3^ Day 0 is 12 h after adding cheese to brine solution.

**Table 8:** Survival of *L. monocytogenes* (log CFU/g) ^1^ in white-brined cheese stored in 10% or 15% NaCl brine at 4, 10, or 24 °C for 91 days in the absence or presence of *L. reuteri* inoculated into milk, brine solution, or distilled water.

Storage Time (Days)									
Treatments ^2^	0 ^3^	3	7	14	21	28	49	71	91
T1	4.80 ± 0.11 ^Ca^	6.09 ± 0.09 ^ABbc^	5.86 ± 0.08 ^ABCDEFb^	6.28 ± 0.17 ^JKcd^	6.02 ± 0.18 ^Gbc^	6.50 ± 0.41 ^Ld^	6.80 ± 0.13 ^Ne^	6.46 ± 0.02 ^Ld^	6.91 ± 0.05 ^Ie^
T2	4.81 ± 0.07 ^Ca^	6.12 ± 0.03 ^ABd^	5.96 ± 0.07 ^CDEFcd^	5.34 ≠ 0.09 ^EFb^	5.97 ± 0.54 ^FGcd^	5.62 ± 0.40 ^FGHIJbc^	5.73 ± 0.07 ^IJKcd^	5.31 ± 0.09 ^HIb^	5.93 ± 0.16 ^Fcd^
T3	4.67 ± 0.05 ^BCb^	6.20 ± 0.06 ^ABCf^	5.45 ± 0.01 ^ABe^	5.13 ± 0.13 ^CDEd^	4.93 ± 0.05 ^BCc^	4.47 ± 0.01 ^BCDa^	4.44 ± 0.01 ^BCDa^	4.44 ± 0.02 ^CDa^	4.36 ± 0.07 ^Ba^
T4	4.56 ± 0.13 ^ABCc^	6.10 ± 0.08 ^ABf^	5.40 ± 0.08 ^Ae^	5.26 ± 0.01 ^DEFe^	4.93 ± 0.05 ^BCd^	3.98 ± 0.04 ^ABa^	4.18 ± 0.13 ^Bb^	4.30 ± 0.02 ^Cb^	4.27 ± 0.17 ^Bb^
T5	4.55 ± 0.16 ^ABCa^	6.51 ± 0.39 ^Cd^	6.37 ± 0.44 ^FGcd^	5.94 ± 0.04 ^GHIc^	6.01 ± 0.11 ^Gc^	6.11 ± 0.38 ^IJKLcd^	6.31 ± 0.28 ^LMcd^	5.34 ± 0.11 ^Ib^	6.16 ± 0.11 ^FGHcd^
T6	4.68 ± 0.08 ^BCa^	6.08 ± 0.02 ^ABe^	6.07 ± 0.13 ^DEFGe^	4.93 ± 0.04 ^Cab^	5.31 ± 0.33 ^CDcd^	5.45 ± 0.40 ^EFGHIcd^	5.64 ± 0.23 ^HIJd^	5.14 ± 0.02 ^Gbc^	5.51 ± 0.16 ^Ed^
T7	4.35 ± 0.20 ^Ac^	6.37 ± 0.03 ^BCe^	5.98 ± 0.03 ^CDEFGd^	3.92 ± 0.06 ^Aa^	4.27 ± 0.06 ^Abc^	4.14 ± 0.02 ^ABb^	4.20 ± 0.13 ^Bbc^	4.14 ± 0.03 ^Bb^	4.12 ± 0.04 ^Bbb^
T8	4.42 ± 0.13 ^ABd^	6.37 ± 0.08 ^BCg^	5.85 ± 0.03 ^ABCDEf^	4.43 ± 0.28 ^Bd^	4.14 ± 0.02 ^Ac^	3.82 ± 0.10 ^ABa^	4.09 ± 0.17 ^Bbc^	3.85 ± 0.05 ^Aab^	4.96 ± 0.08 ^CDe^
T9	4.80 ± 0.11 ^Ca^	6.27 ± 0.16 ^ABCbc^	6.07 ± 0.12 ^DEFGb^	6.36 ± 0.03 ^Kbc^	6.27 ± 0.55 ^Gbc^	6.17 ± 0.53 ^JKLb^	6.45 ± 0.15 ^MNbc^	6.73 ± 0.11 ^Mc^	6.37 ± 0.09 ^Hbc^
T10	4.81 ± 0.07 ^Ca^	6.21 ± 0.10 ^ABCc^	6.08 ± 0.16 ^DEFGbc^	5.79 ± 0.06 ^GHb^	4.81 ± 0.20 ^BCa^	4.80 ± 0.50 ^CDEa^	4.75 ± 0.18 ^CDEa^	4.94 ± 0.04 ^EFa^	4.94 ± 0.11 ^CDa^
T11	4.67 ± 0.05 ^ABCd^	6.18 ± 0.16 ^ABCf^	5.47 ± 0.13 ^ABe^	5.48 ± 0.24 ^Fe^	4.52 ± 0.08 ^ABcd^	4.24 ± 0.05 ^ABCab^	4.08 ± 0.07 ^Ba^	4.34 ± 0.05 ^Cbc^	4.37 ± 0.08 ^Bbcd^
T12	4.56 ± 0.13 ^ABCd^	5.92 ± 0.05 ^Ag^	5.37 ± 0.25 ^Af^	5.07 ± 0.06 ^CDe^	4.08 ± 0.04 ^Ac^	3.79 ± 0.01 ^Ab^	3.55 ± 0.17 ^Aab^	3.75 ± 0.05 ^Aab^	3.49 ± 0.18 ^Aa^
T13	4.55 ± 0.16 ^ABCa^	6.26 ± 0.18 ^ABCbc^	6.51 ± 0.46 ^Gcd^	6.38 ± 0.16 ^Kbcd^	6.12 ± 0.40 ^Gbc^	6.36 ± 0.38 ^KLcd^	6.77 ± 0.12 ^Nd^	5.94 ± 0.03 ^JKb^	6.28 ± 0.17 ^GHbc^
T14	4.68 ± 0.08 ^BCa^	6.12 ± 0.03 ^ABcd^	6.34 ± 0.03 ^EFGd^	6.11 ± 0.12 ^IJcd^	5.87 ± 0.51 ^DEFGc^	5.78 ± 0.47 ^GHIJKc^	6.12 ± 0.18 ^KLMdcd^	6.02 ± 0.07 ^Kcd^	5.28 ± 0.22 ^DEb^
T15	4.35 ± 0.20 ^Aa^	6.22 ± 0.05 ^ABCef^	6.36 ± 0.03 ^EFGf^	6.09 ± 0.02 ^IJe^	6.13 ± 0.02 ^Ge^	4.92 ± 0.05 ^CDEd^	4.67 ± 0.12 ^CDEc^	4.57 ± 0.14 ^Dbc^	4.40 ± 0.13 ^Bab^
T16	4.42 ± 0.13 ^ABa^	6.16 ± 0.09 ^ABCde^	6.27 ± 0.05 ^DEFGe^	6.02 ± 0.05 ^HIcd^	5.91 ± 0.06 ^EFGc^	4.93 ± 0.05 ^CDEb^	4.38 ± 0.08 ^BCa^	4.35 ± 0.03 ^Ca^	4.33 ± 0.15 ^Ba^
T17	4.80 ± 0.11 ^Ca^	6.26 ± 0.11 ^ABCc^	6.23 ± 0.14 ^DEFGc^	6.13 ± 0.05 ^IJbc^	5.70 ± 0.14 ^DEFGb^	6.14 ± 0.50 ^JKLbc^	5.99 ± 0.51 ^DJKLbc^	5.78 ± 0.07 ^Kbc^	6.67 ± 0.22 ^Id^
T18	4.81 ± 0.07 ^Ca^	6.18 ± 0.18 ^ABCe^	6.23 ± 0.07 ^DEFGe^	5.42 ± 0.11 ^Fb^	5.41 ± 0.12 ^CDEFb^	5.87 ± 0.09 ^HIJKLcd^	5.70 ± 0.23 ^HIJKDc^	5.97 ± 0.20 ^Kd^	5.89 ± 0.08 ^Fcd^
T19	4.67 ± 0.05 ^BCa^	6.24 ± 0.12 ^ABCe^	5.53 ± 0.10 ^ABCd^	5.41 ± 0.07 ^Fd^	5.12 ± 0.11 ^Cb^	5.17 ± 0.11 ^EFGbc^	5.33 ± 0.06 ^GHIcd^	5.16 ± 0.12 ^GHbc^	5.00 ± 0.08 ^CDb^
T20	4.56 ± 0.13 ^ABCa^	6.01 ± 0.43 ^ABd^	5.38 ± 0.16 ^Ac^	4.88 ± 0.16 ^Cab^	4.98 ± 0.04 ^BCb^	4.97 ± 0.05 ^DEFb^	5.05 ± 0.04 ^EFGbc^	5.06 ± 0.06 ^FGCbc^	4.90 ± 0.19 ^Cab^
T21	4.55 ± 0.16 ^ABCa^	6.17 ± 0.13 ^ABCc^	6.01 ± 0.50 ^CDEFbc^	5.77 ± 0.17 ^Cbc^	5.75 ± 0.18 ^DEFGbc^	5.80 ± 0.19 ^GHIJKbc^	5.52 ± 0.47 ^BHIb^	5.79 ± 0.10 ^Jbc^	6.00 ± 0.21 ^FGbc^
T22	4.68 ± 0.08 ^BCa^	6.10 ± 0.13 ^ABd^	5.92 ± 0.29 ^BCDEFcd^	5.76 ± 0.14 ^Gc^	5.33 ± 0.21 ^CDEb^	5.32 ± 0.23 ^EFGHb^	5.26 ± 0.20 ^FGHb^	5.13 ± 0.03 ^Gb^	5.26 ± 0.26 ^DEb^
T23	4.35 ± 0.20 ^Aa^	6.14 ± 0.12 ^ABCd^	5.86 ± 0.13 ^ABCDEFc^	5.01 ± 0.07 ^Cb^	4.94 ± 0.31 ^BCb^	4.86 ± 0.05 ^CDEb^	4.98 ± 0.03 ^EFb^	4.89 ± 0.11 ^Eb^	4.80 ± 0.11 ^Cb^
T24	4.42 ± 0.13 ^ABa^	6.04 ± 0.10 ^ABd^	5.77 ± 0.16 ^ABCDc^	5.00 ± 0.08 ^Cb^	5.03 ± 0.06 ^BCb^	4.86 ± 0.03 ^CDEb^	4.86 ± 0.06 ^DEFb^	4.82 ± 0.07 ^Eb^	4.74 ± 0.33 ^Cb^

Means from each sampling time in the same column with the same uppercase letters are not significantly different (*p* > 0.05). Means from each sampling treatment in the same row with the same lowercase letters are not significantly different (*p* > 0.05). ^1^ Values are the means of 4 replicates (n = 4) ± SD. ^2^ Treatments as described in Table 1. ^3^ Day 0 is 12 h after adding cheese to brine solution.

**Table 9:** Survival of *L. monocytogenes* (log CFU/mL) ^1^ in cheese brine at 10% or 15% NaCl at 4, 10, or 24 °C for 91 days in the absence or presence of *L. reuteri* inoculated into milk, brine solution, or distilled water.

Day/Treatments ^2^	0 ^3^	3	7	14	21	28	49	71	91
T1	5.62 ± 0.08 ^BCa^	6.19 ± 0.08 ^ABCDabc^	6.01 ± 0.08 ^DEFGHIab^	6.18 ± 0.20 ^Iabc^	6.53 ± 0.92 ^GHbc^	6.61 ± 0.59 ^FGbc^	6.63 ± 0.36 ^Mbc^	6.42 ± 0.06 ^LMbc^	6.21 ± 0.05 ^Labc^
T2	5.60 ± 0.07 ^BCa^	6.16 ± 0.06 ^ABCDc^	6.09 ± 0.12 ^EFGHIc^	5.87 ± 0.09 ^GHb^	6.14 ± 0.25 ^DEFGc^	6.11 ± 0.18 ^Ec^	6.17 ± 0.05 ^Lc^	6.17 ± 0.07 ^Kc^	5.85 ± 0.07 ^Hb^
T3	5.34 ± 0.07 ^Ac^	6.33 ± 0.03 ^ABCDe^	5.83 ± 0.10 ^BCDEFd^	5.21 ± 0.07 ^CDc^	5.20 ± 0.07 ^ABCc^	4.21 ± 0.14 ^Aa^	4.27 ± 0.03 ^CDab^	4.32 ± 0.01 ^CDEab^	4.40 ± 0.07 ^DEb^
T4	5.56 ± 0.17 ^ABCd^	6.24 ± 0.08 ^ABCDe^	5.64 ± 0.06 ^BCDd^	5.24 ± 0.02 ^CDc^	4.91 ± 0.06 ^ABb^	4.24 ± 0.05 ^BCa^	4.29 ± 0.06 ^CDa^	4.21 ± 0.04 ^CDa^	4.38 ± 0.11 ^DEa^
T5	5.45 ± 0.01 ^ABa^	6.07 ± 0.13 ^ABb^	6.13 ± 0.12 ^EFGHIb^	6.53 ± 0.23 ^Jd^	6.64 ± 0.15 ^GHd^	6.44 ± 0.11 ^EFGcd^	6.15 ± 0.29 ^Lb^	6.41 ± 0.05 ^LMcd^	6.21 ± 0.03 ^IJbc^
T6	5.74 ± 0.05 ^Cbc^	6.11 ± 0.03 ^ABCde^	6.25 ± 0.25 ^FGHIe^	6.06 ± 0.05 ^HIede^	5.89 ± 0.29 ^CDEFGcd^	5.61 ± 0.11 ^Dab^	5.86 ± 0.08 ^KLbcd^	5.66 ± 0.07 ^Ibc^	5.40 ± 0.20 ^Ga^
T7	5.70 ± 0.24 ^Cc^	6.31 ± 0.17 ^ABCDd^	6.11 ± 0.07 ^EFGHId^	3.99 ± 0.05 ^Aa^	4.43 ± 0.06 ^Ab^	4.21 ± 0.14 ^BCab^	4.36 ± 0.04 ^CDEb^	4.34 ± 0.04 ^DEb^	4.18 ± 0.01 ^CDab^
T8	5.77 ± 0.04 ^Ce^	6.38 ± 0.06 ^BCDg^	5.97 ± 0.02 ^BCDEFGHf^	4.45 ± 0.02 ^Bd^	4.46 ± 0.01 ^Ad^	4.18 ± 0.01 ^Bc^	3.96 ± 0.04 ^BCa^	4.17 ± 0.03 ^Cc^	4.05 ± 0.05 ^BCb^
T9	5.62 ± 0.08 ^BCa^	6.37 ± 0.08 ^BCDcd^	6.39 ± 0.06 ^GHIcd^	6.55 ± 0.20 ^Jd^	7.40 ± 0.36 ^Ie^	6.16 ± 0.21 ^EFc^	5.81 ± 0.36 ^JKLab^	6.33 ± 0.05 ^Lcd^	6.08 ± 0.04 ^HIbc^
T10	5.60 ± 0.07 ^BCcd^	6.31 ± 0.11 ^ABCDe^	6.32 ± 0.16 ^GHIe^	5.91 ± 0.06 ^GHIde^	5.29 ± 0.14 ^BCbc^	4.67 ± 0.31 ^Ca^	4.62 ± 0.66 ^DEFa^	4.93 ± 0.09 ^Fab^	4.55 ± 0.08 ^Ea^
T11	5.34 ± 0.07 ^Ac^	6.22 ± 0.06 ^ABCDd^	5.23 ± 0.03 ^Ab^	5.31 ± 0.04 ^DEc^	5.31 ± 0.07 ^BCc^	3.99 ± 0.01 ^Bb^	3.71 ± 0.11 ^ABa^	3.90 ± 0.05 ^Bb^	3.86 ± 0.06 ^Bb^
T12	5.56 ± 0.17 ^ABCc^	6.15 ± 0.04 ^ABCDd^	5.57 ± 0.18 ^ABCc^	5.33 ± 0.19 ^DEc^	5.00 ± 0.06 ^ABb^	3.53 ± 0.11 ^Aa^	3.34 ± 0.21 ^Aa^	3.45 ± 0.14 ^Aa^	3.58 ± 0.21 ^Aa^
T13	5.45 ± 0.01 ^ABa^	6.44 ± 0.26 ^CDb^	6.19 ± 0.38 ^EFGHIb^	7.01 ± 0.06 ^Kcd^	7.02 ± 0.34 ^HIcd^	6.61 ± 0.39 ^FGbc^	7.08 ± 0.18 ^Nd^	6.99 ± 0.10 ^Ocd^	6.44 ± 0.19 ^JKb^
T14	5.74 ± 0.05 ^Cb^	6.45 ± 0.03 ^Dc^	6.27 ± 0.22 ^FGHIc^	5.32 ± 0.18 ^DEa^	5.67 ± 0.59 ^BCDEFab^	5.51 ± 0.12 ^Dab^	5.78 ± 0.06 ^Db^	5.78 ± 0.13 ^Ib^	5.46 ± 0.14 ^Gab^
T15	5.70 ± 0.24 ^Cd^	6.20 ± 0.13 ^ABCDe^	6.40 ± 0.11 ^HIe^	5.56 ± 0.10 ^EFcd^	5.46 ± 0.02 ^BCDc^	4.42 ± 0.08 ^BCa^	4.80 ± 0.08 ^GKLb^	4.46 ± 0.01 ^Ea^	4.28 ± 0.02 ^CDa^
T16	5.77 ± 0.04 ^Cc^	6.38 ± 0.04 ^BCDe^	6.46 ± 0.01 ^If^	5.75 ± 0.02 ^FGc^	5.89 ± 0.07 ^CDEFGd^	4.44 ± 0.01 ^BCb^	4.24 ± 0.06 ^CDa^	4.23 ± 0.02 ^CDa^	4.18 ± 0.03 ^CDa^
T17	5.62 ± 0.08 ^BCa^	6.44 ± 0.16 ^Dbc^	5.99 ± 0.50 ^CDEFGHab^	7.16 ± 0.08 ^Kd^	6.58 ± 0.69 ^GHc^	6.84 ± 0.10 ^Gcd^	5.66 ± 0.22 ^IJKLa^	6.84 ± 0.10 ^ANcd^	6.84 ± 0.16 ^Lcd^
T18	5.60 ± 0.07 ^BCa^	6.29 ± 0.23 ^ABCDb^	6.23 ± 0.12 ^EFGHIb^	6.53 ± 0.16 ^Jb^	6.28 ± 0.17 ^EFGHb^	6.33 ± 0.19 ^EFb^	5.75 ± 0.14 ^JKLa^	6.52 ± 0.14 ^Mb^	6.43 ± 0.19 ^JKb^
T19	5.58 ± 0.06 ^BCbcd^	6.19 ± 0.35 ^ABCDe^	5.79 ± 0.11 ^BCDEcde^	5.89 ± 0.06 ^GHIde^	5.49 ± 0.29 ^BCDEabcd^	5.35 ± 0.43 ^Aabc^	5.22 ± 0.15 ^GHIab^	5.15 ± 0.06 ^Gab^	5.04 ± 0.06 ^Fa^
T20	5.56 ± 0.17 ^ABCb^	6.02 ± 0.02 ^Ac^	5.54 ± 0.15 ^ABb^	4.97 ± 0.03 ^Ca^	4.98 ± 0.04 ^ABa^	5.42 ± 0.00 ^Db^	5.05 ± 0.05 ^FGHa^	5.09 ± 0.06 ^Ga^	4.93 ± 0.05 ^Fa^
T21	5.45 ± 0.01 ^ABa^	6.33 ± 0.18 ^ABCDbcd^	6.37 ± 0.23 ^GHIbcd^	6.63 ± 0.32 ^Jd^	6.37 ± 0.55 ^FGHbcd^	6.14 ± 0.21 ^Ebc^	5.42 ± 0.28 ^HIJKa^	5.99 ± 0.08 ^Jb^	6.53 ± 0.15 ^Kcd^
T22	5.76 ± 0.04 ^Cbc^	6.29 ± 0.15 ^ABCDd^	6.29 ± 0.22 ^GHId^	6.61 ± 0.17 ^Je^	5.85 ± 0.30 ^CDEFGc^	5.46 ± 0.01 ^Da^	5.43 ± 0.28 ^HIJKa^	5.35 ± 0.09 ^Ha^	5.50 ± 0.11 ^Gb^
T23	5.65 ± 0.21 ^BCc^	6.24 ± 0.08 ^ABCDd^	6.03 ± 0.05 ^DEFGHId^	4.99 ± 0.07 ^Ca^	5.47 ± 0.01 ^BCDbc^	5.18 ± 0.09 ^Dab^	5.32 ± 0.10 ^GHIb^	5.36 ± 0.03 ^Hb^	5.36 ± 0.30 ^Gb^
T24	5.77 ± 0.04 ^Cc^	6.27 ± 0.02 ^ABCDe^	5.94 ± 0.03 ^BCDEFGd^	5.67 ± 0.15 ^FGc^	5.26 ± 0.05 ^BCb^	5.18 ± 0.01 ^Dab^	5.07 ± 0.04 ^FGHa^	5.11 ± 0.04 ^Ga^	5.10 ± 0.02 ^Fa^

Means from each sampling time in the same column with the same uppercase letters are not significantly different (*p* > 0.05). Means from each sampling treatment in the same row with the same lowercase letters are not significantly different (*p* > 0.05). ^1^ Values are the means of 4 replicates (n = 4) ± SD. ^2^ Treatments as described in Table 1. ^3^ Day 0 is 12 h after adding cheese to brine solution.

## Data Availability

The original contributions presented in this study are included in the article. Further inquiries can be directed to the corresponding authors.
